# Direct genetic transformation bypasses tumor-associated DNA methylation alterations

**DOI:** 10.1186/s13059-025-03650-2

**Published:** 2025-07-17

**Authors:** Sara Hetzel, Eran Hodis, Elena Torlai Triglia, Alexander Kovacsovics, Kathleen Steinmann, Andreas Gnirke, Meiying Cui, Daniel McQuaid, Raha Weigert, Georg Pohl, Mandar D. Muzumdar, Serge Leyvraz, Ulrich Keilholz, Marie-Laure Yaspo, Aviv Regev, Helene Kretzmer, Zachary D. Smith, Alexander Meissner

**Affiliations:** 1 Max Planck Institute for Molecular Genetics, Berlin, Germany; 2 Present Address: Digital Health Cluster, Digital Engineering Faculty, Hasso Plattner Institute for Digital Engineering, University of Potsdam, Potsdam, Germany; 3 Broad Institute of MIT and Harvard, Cambridge, MA USA; 4 Department of Medical Oncology, Dana-Farber Cancer Institute, Boston, MA USA; 5 Mass General Cancer Center, Massachusetts General Hospital, Boston, MA USA; 6 School of Biological and Behavioural Sciences, Queen Mary University of London, London, UK; 7 Department of Genetics, Yale Stem Cell Center, Yale School of Medicine, New Haven, CT USA; 8 Charité Comprehensive Cancer Center, Charité Universitätsmedizin, Berlin, Germany; 9 South San Francisco, Genentech, CA USA

**Keywords:** DNA methylation, Cancer, Epigenetics, Disease models, Genetically engineered mouse models

## Abstract

**Background:**

Tumors represent dynamically evolving populations of mutant cells, and many advances have been made in understanding the biology of their progression. However, there are key unresolved questions about the conditions that support a cell’s initial transformation, which cannot be easily captured in patient populations and are instead modeled using transgenic cellular or animal systems.

**Results:**

Here, we use extensive patient atlas data to define common features of the tumor DNA methylation landscape as they compare to healthy human cells and apply this benchmark to evaluate 21 engineered human and mouse models for their ability to reproduce these patterns.

Notably, we find that genetically induced cellular transformation rarely recapitulates the widespread de novo methylation of Polycomb regulated promoter sequences as found in clinical samples, but can trigger global changes in DNA methylation levels that are consistent with extensive proliferation in vitro.

**Conclusions:**

Our results raise pertinent questions about the relationship between genetic and epigenetic aspects of tumorigenesis as well as provide an important molecular reference for evaluating existing and emerging tumor models.

**Supplementary Information:**

The online version contains supplementary material available at 10.1186/s13059-025-03650-2.

## Background

Tumors are classically described as a collection of genetically driven diseases that emerge through a series of mutagenic events over many years. For the past four decades, this prevailing view has been complemented by a growing body of research on epigenetic alterations—from local changes in DNA methylation to broad reprogramming of nuclear topology—which emerge as distinct signatures in patient cohorts [1]. For example, it is well established that CpG island (CGI)-containing promoters become selectively hypermethylated in many tumor types, particularly those that are canonically regulated by Polycomb repressive complex (PRC) 2 during normal development and maintained in an unmethylated state within healthy adult cells [2–8]. At the same time, tumors often lose methylation globally across gene-poor megabase-scale regions termed partially methylated domains (PMDs), which are also subject to varying degrees of hypomethylation during aging and long-term culture [8, 9]. Despite the massive amount of data supporting these observations, very little is known about what specific purpose these patterns may serve in tumor biology, including details regarding how they are established or participate in progression.

Tumor biopsies, xenografts, cancer cell lines, and organoid models are all derived from patient cells that already carry a complex history of genetic and epigenetic adaptations. As such, efforts to investigate the initial somatic-to-tumor transition largely rely on genetically engineered mouse models and human cell lines [10]. However, it has been observed that many in vivo models of solid tumors do not recapitulate the full pathobiology of their respective diseases, while in vitro models provide only limited phenotypic read outs [11–13]. The continued expansion and ongoing innovation of these models therefore requires careful molecular benchmarking, particularly given the degree to which epigenetic aspects of cellular identity have been used to inform concepts related to phenotypic plasticity, therapeutic resistance, immune tolerance, and tumor evolution.

Epigenetic changes such as CGI hypermethylation are well established across diverse cancer types, and previous efforts have enabled identification of biomarkers that stratify tumor from healthy samples, distinguish different tumor (sub-)types, or provide context related to the cell of origin [14–20]. Other studies have investigated single-tumor or pan-cancer DNA methylation dynamics as downstream components of complex gene regulatory networks [21–25]. Despite the incredible value of these datasets to better understand the genetic and epigenetic components of cancer biology, a comprehensive investigation of how well these features are captured by transgenic models has not been conducted. Additionally, the degree to which tumor-like CGI signatures may already be found in healthy tissues has also not been definitively determined, even though knowing the diversity of pre-existing CGI methylation within the human body would ensure a robust assessment of tumor-like DNA methylation dynamics in model systems from different sources and assays.

To comprehensively investigate how well genetically engineered cancer models mirror molecular features seen in patients, we first collected thousands of publicly available clinical and healthy reference data sets across a broad range of human tumor types and supplemented these with newly generated whole-genome bisulfite sequencing (WGBS) data. We then used this cohort to build a comprehensive benchmark of tumor type-specific and pan-cancer signatures and compared it to a diverse atlas of healthy, purified human cell types. Next, we selected two well-characterized human in vitro models and 19 in vivo mouse models to explore the extent to which bona fide genetic drivers induce the typical epigenetic changes found in patients. By examining these models through the lens of patient signatures, we conclude that essentially none trigger the widespread changes in promoter methylation that are found in human tumors. Instead, in vitro models capture proliferation-induced changes in global methylation, a cancer-like feature that nonetheless differed from the patterns found in patient samples. Within mice, only models of T cell acute lymphoblastic leukemia (T-ALL) partially gained promoter methylation, even when using genetic drivers that failed to cause equivalent changes in other systems, suggesting a cell type rather than driver-specific effect. Our analyses highlight a valuable opportunity to build more accurate models that capture common epigenetic features of human tumors, and to apply these tools to study the specific role of this signature as it interacts with genetic drivers.

## Results

### CGI hypermethylation across human tumors

**Fig. 1:**
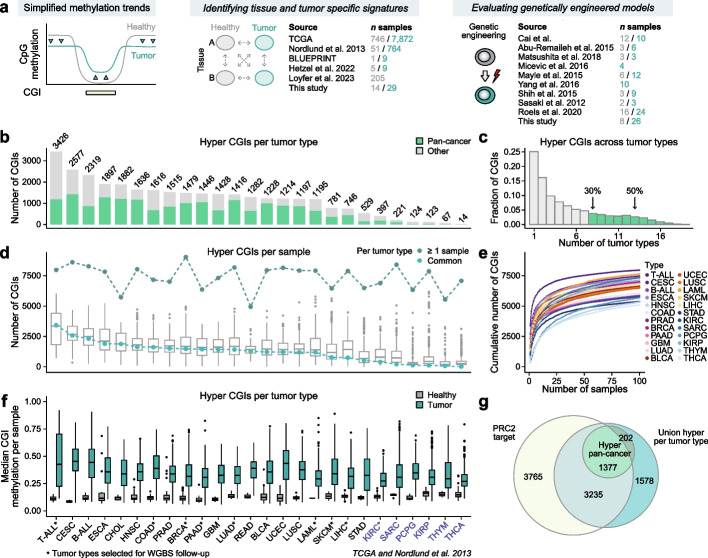
Identification of pan-cancer CGI hypermethylation signatures. **a** Schematic of the general DNA methylation trends that distinguish primary tumors from somatic cells, including global loss and focal gain at CpG islands (CGIs). The extent to which different genetically engineered experimental models recapitulate this genome-scale transformation has not been systematically investigated and is the subject of this study. For this purpose, a benchmark of DNA methylation dynamics within and across tumor and healthy cell types is required in order to assess the robustness of the detection of tumor-specific signatures. Additional schematics are included to summarize the data sets collected or generated to relate clinical signatures to experimental models. **b** Number of hyper CGIs commonly found (≥ 50% of patients) for each of the 26 tumor types. Tumor types examined (see labels in Fig. 1f): T cell acute lymphoblastic leukemia (T-ALL), cervical squamous cell carcinoma and endocervical adenocarcinoma (CESC), B cell acute lymphoblastic leukemia (B-ALL), esophageal carcinoma (ESCA), cholangiocarcinoma (CHOL), head and neck squamous cell carcinoma (HNSC), colon adenocarcinoma (COAD), prostate adenocarcinoma (PRAD), breast invasive carcinoma (BRCA), pancreatic adenocarcinoma (PAAD), glioblastoma (GBM), lung adenocarcinoma (LUAD), rectum adenocarcinoma (READ), bladder urothelial carcinoma (BLCA), uterine corpus endometrial carcinoma (UCEC), lung squamous cell carcinoma (LUSC), acute myeloid leukemia (LAML), skin cutaneous melanoma (SKCM), liver hepatocellular carcinoma (LIHC), stomach adenocarcinoma (STAD), kidney renal clear cell carcinoma (KIRC), sarcoma (SARC), pheochromocytoma and paraganglioma (PCPG), kidney renal papillary cell carcinoma (KIRP), thymoma (THYM), thyroid carcinoma (THCA). Blue labels indicate tumor types that are less prone to CGI hypermethylation. **c** Histogram showing the fraction of common hyper CGIs that are shared across an increasing number of different tumor types. CGIs that are called in at least 30% of single tumor type comparisons were assigned to the pan-cancer hyper CGI set (marked in green). **d** Boxplot showing the number of hyper CGIs that are called per patient, with the number of total hyper CGIs found in at least one patient or included in the “common” set highlighted (upper and lower line, respectively). Six tumor types show comparatively low per-sample hyper CGI signal. For these, the number of common hyper CGIs called using our approach is below the core distribution of hyper CGIs for single samples (≤ 25% quantile), suggesting that these cancer types may be less prone to systematic CGI hypermethylation as part of their pathobiology. Lines denote the median, edges denote the interquartile range (IQR), whiskers denote 1.5 × IQR and minima/maxima are represented by dots. **e** Saturation analysis of the number of hyper CGIs cumulatively observed from random samples of 100 patient tumors (see Methods). A large fraction of each tumor type's overall CGI methylation profile is already captured by a comparatively small number of patient samples (< 25). **f** Boxplot showing the median methylation of each common tumor type-specific hyper CGI set for healthy and tumor samples. Note that each comparison uses its own set of commonly hypermethylated CGIs as called for the indicated tumor type. Lines denote the median, edges denote the interquartile range (IQR), whiskers denote 1.5 × IQR and minima/maxima are represented by dots. **g** Overlap between PRC2 target, pan-cancer hyper and the union of tumor type-specific hyper CGIs

Our main motivation was to study the emergence and drivers of DNA methylation alterations within experimental models (Fig. 1a). To do so, we first had to establish the baseline expectation for the diversity of patient signatures (Fig. 1a). For this purpose, we took advantage of data generated through The Cancer Genome Atlas (TCGA) as well as publicly available acute lymphoblastic leukemia samples to quantify the frequency and levels of CGI hypermethylation across 26 tumor types and their corresponding healthy tissue (Fig. 1b, n = 9433 samples, 450 k methylation arrays, Additional file 1: Table S1). For each tumor type, we defined a set of commonly hypermethylated (hyper) CGIs that are hypermethylated in at least 50% of the samples compared to the corresponding healthy control signature. In this manner, we capture the most consistent clinical changes, even in heterogeneous cases where a single category contains many subtypes.

The number of commonly hypermethylated CGIs varied substantially across tumor types, ranging from more than 3000 CGIs in T-ALL to only 14 CGIs for thyroid carcinoma, and showed varying degrees of overlap (Fig. 1b and c, Additional file 1: Table S2). Notably, all show a clear enrichment for PRC2-based regulation in human embryonic stem cells (hESCs) in line with previous work [6] (Additional file 2: Fig. S1a). In all tumor types, the total number of CGIs that show hypermethylation in at least one sample is much larger than the common set (median sixfold larger), likely reflecting the stringent nature of our intersection-based approach as it compares with patient-level heterogeneity (Fig. 1d). The somewhat individualized nature of single tumor profiles nonetheless appears to conform to a general CGI hypermethylation landscape. For example, for each tumor type, we find that a large fraction of observed CGI targets are called as hypermethylated after sampling a small number of patient biopsies, supporting a model where these patterns are broadly constrained (Fig. 1e). The number of common hyper CGIs per tumor type is also representative of the number of hyper CGIs in individual samples, suggesting that number, identity and methylation level of these CGIs reflect core properties of each individual disease (Fig. 1d–f).

Out of the 26 tumor types that we examined, six had comparatively small numbers of commonly hypermethylated CGIs (≤ 25% quantile of single samples), which suggests a higher variability in hypermethylation targets between individuals: kidney renal clear cell carcinoma (KIRC), sarcoma (SARC), pheochromocytoma and paraganglioma (PCPG), kidney renal papillary cell carcinoma (KIRP), thymoma (THYM), and thyroid carcinoma (THCA) (Fig. 1d). Individual tumor samples within these cohorts also had fewer hyper CGIs overall, suggesting that their progression may be less reliant or prone to CGI methylation. Based on the available public data, we can exclude tumor purity as the underlying reason (Additional file 2: Fig. S1b). Interestingly, in the case of KIRC and KIRP, variation in CGI methylation seems to relate directly to clinical stage, where only late-stage kidney cancers show strong signatures per sample (Additional file 2: Fig. S1c). The acquisition of CGI methylation only at later clinical stages appears to be a phenomenon specific to kidney cancer, as all others show a relatively comparable number of hypermethylated CGIs across stages (Additional file 2: Fig. S1c and d).

We found that 1579 CGIs are commonly targeted (≥ 30%) among the examined tumor types, a set that we term “pan-cancer hyper CGIs” (see Methods, Fig. 1b,c and g, Additional file 2: Fig. S1e). These CGIs are enriched in the promoters of genes associated with the neural lineage, which might be related to the underrepresentation of neural tumor types in the cohort and the correspondingly high likelihood that these promoters are silenced by PRC2 in the majority of tissues considered here (Additional file 2: Fig. S1f). Finally, in addition to CGI hypermethylation, tumor types display varying degrees of global hypomethylation (Additional file 2: Fig. S1e and g, approximated by measuring isolated “solo-WCGW CpGs” as reported previously [9]). Global loss of methylation is most pronounced in colorectal, bladder and liver carcinoma and virtually absent in acute leukemias [26], thymoma and thyroid carcinoma (Additional file 2: Fig. S1e).

Taken together, our analysis establishes a clear pan-cancer description of characteristic DNA methylation patterns seen across the majority of tumor types. While previous studies have reported similar trends for individual tumors or subsets [15–19, 21–25], this type of comprehensive reference atlas allowed us to generalize these features across 26 tumor types and set a clear molecular benchmark to evaluate different human and mouse models.

### CGI methylation in adult cell types and the relationship to the tissue of origin

Previous reports have shown that CGI hypermethylation occurs in a tumor type-specific manner and that even tumor subtypes can be distinguished based on their DNA methylation profile [15, 16, 18]. This property has been linked to the cell or tissue of origin and can be molecularly explained by the observation that CGI hypermethylation is strongly associated with PRC2 recruitment in healthy tissues: cell type-specific promoters will not be methylated in a given tissue if they are actively transcribed, but will be repressed and susceptible to hypermethylation in other tissues [18]. Ultimately, we find that this connection between PRC2 regulation in healthy cells and DNA methylation in cancer leads to an increased overlap of hyper CGIs across tumor types based on the proximity of their tissues of origin (Additional file 2: Fig. S2a).

For this reason, it may seem crucial to use precisely matching healthy tissue as a comparison for each tumor type, but these might not always be available due to uncertainty about the cell of origin or the lack of data from rare, purified cell types. In order to confirm that tumor-specific patterns are reflective of transformation itself, we made use of a recently published WGBS cohort of 46 sorted human cell types (205 samples) [27]. For our tumor hyper CGI definition (Fig. 1), we only considered CGIs that were unmethylated in the respective healthy tissue (mean methylation ≤ 0.2). This applies to most CGIs within purified cells, while about a third are methylated (8973 out of the 26337 CGIs with a mean methylation > 0.2). As expected, these methylated CGIs are overwhelmingly shared across tissues (on average 90% are shared between two cell types) and are generally found in gene bodies or intergenic regions rather than within promoter regions (Additional file 2: Fig. S2b and c).

**Fig. 2:**
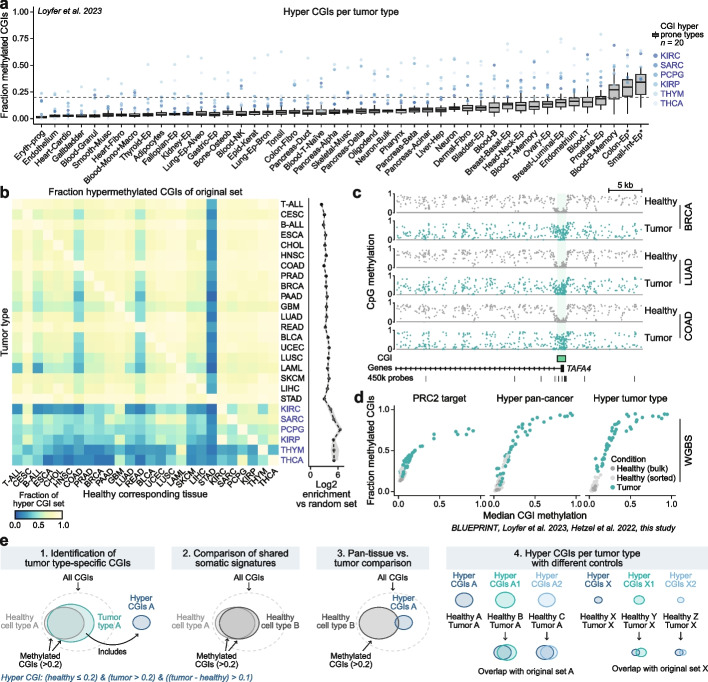
Cancer-associated CGI hypermethylation is not observed in most healthy cell types. **a** Boxplot displaying the fraction of each tumor type-specific hyper CGI set that is called as methylated across a panel of 46 purified human cell types. Each data point represents the overlap between the methylated CGIs within a given healthy cell type and each tumor type’s common hyper CGI set to highlight the degree to which healthy human cells carry cancer-like methylation patterns (denoted as boxplot or dots). Tumor types with overall low numbers of hyper CGIs are flagged as exceptions for reasons described in text. Additionally, three healthy cell types show a higher degree of cancer-associated CGI methylation, including memory B cells, colon and small intestinal epithelium. Memory B cells have been reported to show elevated CGI methylation levels [28, 29], while colon and small intestine epithelium (*) show higher levels but were obtained as part of tumor resections from cancer patients or from patients with other intestinal diseases, which confounds our ability to say that these cells definitively represent a true “healthy” sample [27]. Lines denote the median, edges denote the IQR and whiskers denote 1.5 × IQR. Eryth-prog = erythrocyte progenitor, Cardio = cardiomyocyte, Granul = granulocyte, Musc = muscle, Fibro = fibroblast, Mono = monocyte, Macro = macrophage, Ep = Epithelium, Alveo = alveolar, Osteob = osteoblast, NK = natural killer cell, Epid-Kerat = epidermal keratinocyte, Bron = bronchus, Oligodend = oligodendrocyte, Hep = hepatocyte, Small-Int = small intestine. **b** Comparison between tumor type-specific hyper CGIs and different control tissues. Left: Heatmap displaying the fraction of each tumor type’s original hyper CGI set (defined with the correct control) that are called when using any other healthy tissue as the control sample (median fraction recovered = 0.8). The diagonal is always 1, as the complete CGI set can be found when compared against the correct control tissue (*x*-axis = control tissue, *y*-axis = tumor). Right: Log2-transformed enrichment of hyper CGIs called compared to random sampling (0 reflects no difference to random sampling). Dots denote the median and the gray area denotes the IQR. **c** Genome browser track of the *TAFA4* locus with exemplary whole genome bisulfite sequencing (WGBS) samples generated from healthy breast, lung and colon tissue as well as from primary tumors derived from these tissues. The tumor samples exhibit hypermethylation of the promoter CGI and loss of methylation in inter- and intragenic regions. **d** Scatterplot showing the median methylation and fraction of methylated CGIs for PRC2 target CGIs, as well as for the pan-cancer and tumor type-specific hyper CGI sets for healthy and tumor WGBS samples. **e** Summary of CGI methylation dynamics in healthy cells as they relate to tumors: Approximately one third of all CGIs are methylated in healthy tissues (1) and most of them overlap across sampled cell types (2). Tumor types methylate additional CGIs as part of their biology, which rarely overlap with signatures found in any healthy cell type (3). This finding is supported by the fact that a large fraction of tumor-associated hyper CGIs can be recapitulated when using different control tissues (4, left). The main exceptions to this are tumor types with overall low numbers of hyper CGIs (4, right). Ovals are not drawn in a quantitative manner, but to roughly reflect the size of CGI sets

When connecting our observations from the static somatic methylation state to the tumor space, we find that tumour-specific hyper CGIs are rarely called as methylated across the majority of healthy tissues (Fig. 2a, Additional file 2: Fig. S2d; exception being the six tumor types that display the fewest number of hypermethylated CGIs). Conversely, the only cell types from our healthy set with higher proportions of methylated CGIs are the colon and small intestinal epithelium, as well as memory B cells. Although it has been shown that memory B cells can display higher overall CGI methylation than other tissues [28, 29], it should be noted that the intestinal samples were taken from patients with intestinal tumors and other disease states. This confounder affects our ability to discern whether cancer-like CGI methylation signatures are common to healthy intestinal epithelium, reflective of a precancerous or other disease state, or represent contamination with infiltrating tumor tissue [27, 30–34].

Overall, our intersectional analyses between diverse tissues and tumors strongly support the conclusion that CGI hypermethylation is an acquired feature of transformation, and not a latent signature within control samples. In line with this observation, we recover the majority of tumor type-specific hypermethylated CGIs even when using unmatched control tissues for our TCGA-based analysis, which in many cases only contributes a small fraction of additional false positive targets (Fig. 2b, Additional file 2: Fig. S3a). To generalize our observations, we used newly generated WGBS samples of seven different tumor types as well as publicly available acute leukemia samples to verify that our array-based hyper CGI sets can distinguish healthy and tumor samples. For tumor types with strong hypermethylation signatures, we could readily separate tumor from normal by evaluating our pan-cancer CGI set as well as by considering all PRC2 target CGIs as identified in hESCs (Fig. 2c and d, Additional file 2: Fig. S3b-e, Additional file 1: Table S3). For tumor types with less pronounced signatures like KIRC, only the tumor type-specific hyper CGI set could distinguish healthy and tumor samples (Additional file 2: Fig. S3b). Additionally, we found that our WGBS data sets exhibited similar global methylation dynamics as approximated using the 450 k array cohort (Additional file 2: Fig. S3e). These observations indicate that the identified tumor methylation signatures are robust to different DNA methylation assays.

Taken together, our analyses show that a large fraction of broad tumor type-specific CGI hypermethylation effects can be robustly detected even when using very different reference tissues, highlighting the extent to which this departure in CGI regulation is typical of transformation and rarely observed in the biology of healthy tissues (Fig. 2e). Nevertheless, the matching cell or tissue of origin represents the optimal control tissue for the analysis of tumor-specific DNA methylation changes, especially when considering more focused dynamics. Ultimately, these results argue that CGI methylation is a bona fide feature of human tumorigenesis, even if it is distributed in tumor type-specific patterns.

### Genetically engineered melanoma cells do not acquire CGI hypermethylation

With these insights, we next assessed DNA methylation changes in genetically engineered cancer models by investigating human in vitro systems, in particular a recently reported 2D culture model of skin cutaneous melanoma (SKCM) that starts with healthy primary human melanocytes and serially introduces a number of disease relevant mutations over months in culture [35]. Here, consecutive knock-in or knock-out mutations dysregulate key pathways, including RB (via *CDKN2A* knock-out), MAPK (via *BRAF* V600E mutation), telomerase (via *TERT* promoter mutation), PI3K/AKT (via knock-out of *PTEN*), p53 (via knock-out of *TP53*), and Wnt (via knock-out of *APC*), leading to the formation of highly proliferative cells that form tumors in NOD-SCID gamma mice [35]. As this model demonstrated features of malignancy associated with defined genetic events, such as WNT activation leading to spontaneous visceral metastasis [35], we reasoned that it would provide a powerful platform to address how genetic alterations might relate to CGI methylation as well as the potential dependence of these patterns on proliferation or transplantation.

**Fig. 3:**
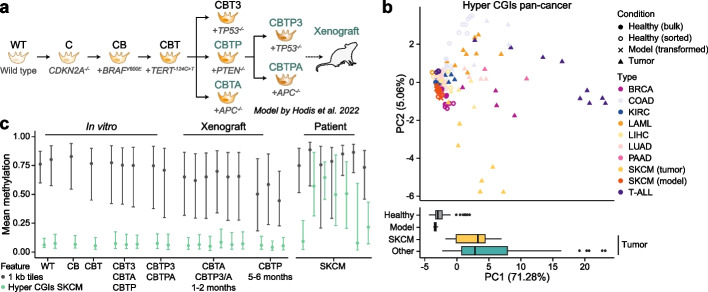
Absence of CGI hypermethylation in in vitro models of melanoma. **a** Schematic of the melanoma model introduced by Hodis et al. (Ref. [35]). Wild type (WT) melanocytes were sequentially edited to generate nine different cell lines to model genetic drivers of melanoma. Edits included the commonly mutated genes *CDKN2A* (C), *BRAF* (B), *TERT* (T), *TP53* (3), *PTEN* (P) and *APC* (A). Cultured models marked in green were additionally used to generate xenograft models. **b** Top: Principal component analysis (PCA) of healthy, tumor and engineered melanocyte model samples based on pan-cancer hyper CGI methylation. Samples of the melanoma model group closely to healthy tissues but not the majority of tumor samples. Bottom: Boxplot showing the distribution of healthy, engineered melanocyte model and tumor (split by melanoma and other) samples on the first principal component. Lines denote the median, edges denote the IQR, whiskers denote 1.5 × IQR and minima/maxima are represented by dots. **c** Distribution of the mean methylation of 1 kilobase (kb) tiles and commonly hypermethylated CGIs (SKCM) across the different melanoma models and replicates. Lines denote the IQR and dots denote the median

**Fig. 4:**
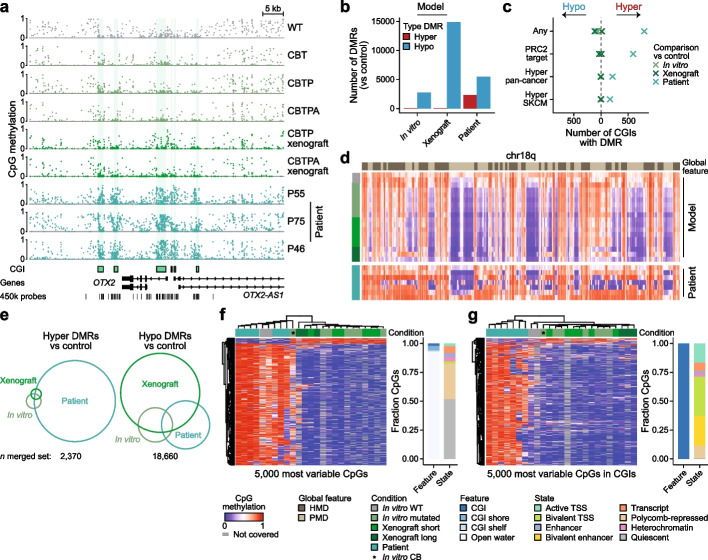
Melanoma patient signatures differ from experimentally transformed melanocytes. **a** Genome browser track of the *OTX2* locus with exemplary WGBS samples of cultured wild type and mutated melanocytes, resulting xenografts as well as patient melanomas. While the tumor samples show different degrees of CGI hypermethylation, the melanocyte model samples maintain CGIs in an unmethylated state. **b** Barplot showing the number of hyper- and hypomethylated differentially methylated regions (DMRs) called between in vitro model, xenograft or patient and control samples. **c** Comparison of the number of CGIs that overlap hyper- or hypomethylated DMRs between control and model or patient samples, split according to distinct features (all, PRC2-regulated, pan-cancer, and defined for SKCM specifically). Only the patient samples display a detectable number of hypermethylated CGIs for any category. **d** Heatmap showing the DNA methylation landscape of chromosome 18q averaged across 100 kb tiles for wild type and knockout in vitro melanocytes, xenografts and melanoma patients. The annotation of highly and partially methylated domains (HMDs, PMDs) was defined according to the wild type melanocyte samples (see Methods). **e** Overlap between hyper- or hypomethylated DMRs of the three different experimental comparisons (in vitro model, xenografts or patients) against control. In order to compare the overlap, we merged DMR sets from all comparisons (separated by hyper and hypo) and calculated the overlap between individual sets. **f** Left: Heatmap and hierarchical clustering of the 5000 most variable CpGs across the melanoma cohort. Patient, wild type melanocytes and one mutated melanocyte sample (CB) cluster separately from the more hypomethylated model samples. Right: Distribution of most variable CpGs in CGI-related features and chromatin states (based on penis foreskin melanocytes). The majority of CpGs are found in quiescent, heterochromatic or Polycomb-repressed open water regions and rarely overlap features with clear regulatory functions. **g** Same as in **f** but based on the 5000 most variable CpGs within CGIs. Here, patient samples cluster separately from all model samples (wild type, knockout, xenograft) and CpGs are frequently found in bivalent domains

Importantly, our previous evaluation confirmed that skin cutaneous melanoma (SKCM) displays the typical tumor CGI hypermethylation patterns (Fig. 1b, Additional file 2: Fig. S1a-e). Therefore, we generated WGBS data from nine different stages of the melanoma model, including most in vitro stages as well as in vivo tumor xenografts (Fig. 3a, Additional file 1: Table S3). A principal component analysis based on pan-cancer hyper CGIs revealed that all model samples, including wild type melanocytes, grouped closely with healthy tissue samples rather than with melanoma (or most other) patient tumors (Fig. 3b). We then inspected the set of commonly hypermethylated SKCM CGIs and again found no evidence of melanoma-specific CGI hypermethylation in any of the mutant genotypes, including after xenotransplantation (Figs. 3c and 4a, Additional file 2: Fig. S4a and b). Notably, some of the transformed melanocyte models are cultured for hundreds of days prior to sampling and exhibit a global loss of methylation—likely as a function of prolonged proliferation—demonstrating that such changes can be decoupled from de novo methylation of CGI-containing promoters [9, 36] (Fig. 3c, Additional file 2: Fig. S4a).

To assess whether we could detect more subtle or specific CGI methylation changes within the melanocyte model, we performed differentially methylated region (DMR) calling between in vitro, xenograft or patient samples and three control samples, respectively. For the controls, we combined two wild type melanocytes and the earliest, not yet immortalized in vitro sample (CB) that still looked very comparable to the healthy counterparts genome-wide (Additional file 2: Fig. S5a). While we detected 2320 hypermethylated DMRs in patient tumors relative to control samples, we found fewer than 80 hypermethylated DMRs when we evaluated either the in vitro models or their xenografts (Fig. 4b, Additional file 2: Fig. S5b). Consistent with this observation, CGIs that overlap with hypermethylated DMRs share the same biological enrichments as our pan-cancer hyper CGI set, are almost exclusively hypermethylated in patients, and show limited responsiveness within the genetic models (Fig. 4c, Additional file 2: Fig. S1f, 5c and d).

In contrast to the patient-specific nature of CGI methylation, model samples (especially the xenografts) are mainly characterized by a large number of hypomethylated DMRs that likely reflect proliferation-dependent loss of genome-wide DNA methylation (Fig. 3c, 4b). As reported previously, loss of global methylation occurs preferentially in late-replicating, gene-poor PMDs, and is often more extreme over prolonged culture compared to patient tumors [37] (Fig. 4d). Within the in vitro samples and their xenografts, PMD methylation loss appears to occur across consistent regions and correlates with their proliferative histories. In contrast, these regions are far more variable across the patient samples, including differences in their genomic localization as well as methylation levels (Fig. 4d and e). Beyond this observation, it was not straightforward to understand whether differences in PMD methylation patterns between the model and patient samples indicate an overall higher proliferative history in the melanocyte model or a more general experimental artifact. Numerous epigenetic scores have been developed to estimate mitotic age by tracking either hyper- or hypomethylation events, two phenomena that are generally linked in human patients but uncoupled in the experimental model. Thus, our efforts to apply these scores led to conflicting results: those based on global methylation loss estimated a higher mitotic age for the model, while those based on de novo methylation estimated a higher mitotic age for patient samples (Additional file 2: Fig. S5e).

Finally, unsupervised clustering based on the 5000 most variably methylated CpGs across our dataset also shows a clear separation between patient tumors and in vitro models. When sampled from the entire genome, variable CpGs are largely located outside of regulatory features and group patient samples with wild type (as well as CB) melanocyte samples, which cluster away from the less methylated, transformed in vitro and xenograft samples (Fig. 4f). In contrast, when considering the 5000 most variable CpGs within CGIs, patient tumors separate from all model samples, with the corresponding CpGs again highly enriched for bivalent domains (Fig. 4g). Collectively, these results suggest that the introduction of tumor-associated genetic alterations in vitro is not sufficient to trigger robust CGI hypermethylation in melanocytes. Furthermore, these signatures also do not appear after transplantation in immunocompromised mice, at least for the described model.

### Effects of senescence and genetic transformation in human fibroblasts

**Fig. 5:**
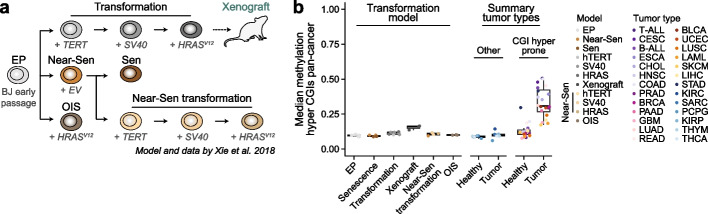
BJ fibroblast transformation models do not induce widespread CGI hypermethylation. **a** Schematic of the senescence and transformation model utilized by Xie et al. (Ref. [39]). BJ fibroblasts were transduced either sequentially with human telomerase catalytic subunit (TERT), the simian virus 40 large T antigen (SV40), and the H-Ras oncoprotein (HRAS, with potential subsequent implantation into a xenograft model) or with an empty vector (EV) that over time served as a model for senescence (Sen). Additionally, oncogene-induced senescence (OIS) was achieved by transfecting the cells directly with the H-Ras oncoprotein. **b** Boxplot showing the median methylation across pan-cancer hyper CGIs for samples of the BJ senescence and transformation model as well as for individual tumor types (450 k array, split by types prone to CGI hypermethylation and exceptions from Fig. 2). Neither transformation nor senescence models show notable CGI hypermethylation, while transformed cells that were implanted into immunocompromised mice exhibit a mild gain. Lines denote the median, edges denote the IQR, whiskers denote 1.5 × IQR and minima/maxima are represented by dots

To further investigate whether other cancer models display the hypermethylation signatures we identified in patients, we revisited a classic model initially reported by Weinberg and colleagues in 1999, which introduces human telomerase catalytic subunit (*hTERT*), the simian virus 40 large T antigen (SV40), and the H-Ras oncoprotein (*H-RAS*^*V12*^) into BJ fibroblasts [38]. Recently, the same model was used to specifically investigate epigenetic differences between senescence and transformation [39] (Fig. 5a). When we globally compare published DNA methylation data from the latter study with our assembled cohort of patient samples, the overall outcome is quite distinct from the hypermethylation observed in human tumors: only 3% of probes gain DNA methylation upon transformation with a difference > 0.1 between average HRAS transformed and early passage wild type replicates (Fig. 5b, Additional file 2: Fig. S6a, Additional file 1: Table S4). Overall, samples do not deviate from ranges seen in healthy cells, though we did note a slight increase in the median methylation in transplanted xenografts that appears to extend to the same PRC2-targeted and pan-cancer hyper CGIs found in patients (Fig. 5b, Additional file 2: Fig. S6b, see Methods). It is, however, worth highlighting that BJs are extensively cultured and effectively immortalized prior to the onset of the experiment, such that the starting cell population (EP) already exhibits physiologically abnormal global DNA hypomethylation levels (Additional file 2: Fig. S6a). Finally, although this classic experimental model does not have an analogous tumor type, our comparisons between the human tissue atlas and patient samples show that CGI hypermethylation is generally robust to cross-tissue comparisons, supporting the conclusion that introduction of classic oncogenes is not sufficient to induce clinical signatures of de novo CGI methylation in human fibroblasts (Fig. 2b).

### Patient-like CGI hypermethylation is rarely observed in transgenic mouse models

To complement cellular transformation models, genetically engineered mice are widely used to study the emergence of tumors in vivo. In order to assess the degree of hypermethylation acquired in these contexts, we assembled a large cohort of newly generated and publicly available data sets for 19 mouse models spanning seven tumor types. We generated reduced representation bisulfite sequencing (RRBS) data sets for two commonly used colon adenoma/carcinoma models (*Apc*^*Min/*+^ and *Apc*^*fl/*+^ combined with different *Kras* mutations (*Kras*^*G12D/*+^*, Kras*^*G13D/*+^ or *Kras*^*A146T/*+^)) [40–42] as well as target enrichment methylation data sets for a pancreatic adenocarcinoma model (driven by *Kras*^*G12D/*+^) that can be further accelerated by genetic obesity (KCO model) [43]. Additionally, we incorporated previously published data sets of a breast cancer model (MMTV-PyMT) [44], a chemically induced colon cancer model (AOM/DSS treatment) [44], a spontaneous liver tumor model (C3H strain liver tumors) [40], two melanoma models (*Braf*^*V600E*^/*Pten*^*fl/fl*^ and *Braf*^*V600E*^/*Pten*^*fl/fl*^*/Dnmt3b*^*fl/fl*^) [45], as well as a variety of acute myeloid leukemia (LAML) (*Dnmt3a*^*fl/fl*^*, Flt3-ITD*^*KI*^*, Flt3-ITD*^*KI*^*/Dnmt3a*^*fl/*+^*, Tet2*^*fl/fl*^*/FLT3-ITD, Idh1(R132H)*^*KI*^) [46–49] and T-ALL models (*Dnmt3a*^*fl/fl*^*, FLT3-ITD* (retroviral infection),* FLT3-ITD/Dnmt3a*^*fl/fl*^*, Pten*^*fl/fl*^*, CD2-Lmo2*^*tg*^) [46, 47, 50].

**Fig. 6:**
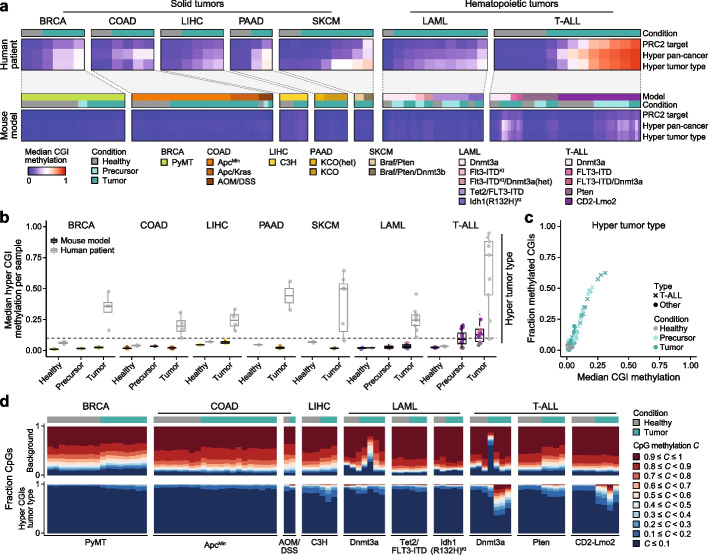
CGI hypermethylation is rarely observed in experimental mouse models. **a** Heatmaps showing the median methylation of PRC2 target, pan-cancer and tumor type-specific hyper CGIs in human patients (top) and a broad selection of genetic, chemical or spontaneous mouse tumorigenesis models (bottom). PRC2 targets were defined separately based on human and mouse annotations; hyper CGI sets were defined by identifying similar CGIs between both species that overlapped the human hyper CGI sets (see Methods). While the patient samples show varying degrees of CGI hypermethylation, only T-ALL mouse models exhibit any notable gain of methylation at these loci. Human patients were profiled using WGBS, while publicly available and newly generated mouse models were profiled using different assays (WGBS: AOM/DSS; PRC2 target enrichment: KCO(het) and KCO; enhanced reduced representation bisulfite sequencing (ERRBS): PyMT, *Tet2*^*fl/fl*^*/FLT3-ITD* and *Idh1(R132H)*^*KI*^; RRBS: all others). Precursor refers to the following: Hyperplasia for PyMT; DSS treatment (colitis) for AOM/DSS; myelodysplastic syndrome or another pre-leukemic state for LAML; *Pten* knockout cells or cells overexpressing Lmo2 prior to actual disease state for T-ALL. **b** Boxplot showing the median methylation across tumor type-specific hyper CGIs for mouse models (black box) and human patients (gray box). Lines denote the median, edges denote the IQR, whiskers denote 1.5 × IQR and minima/maxima are represented by dots. **c** Scatterplot showing the median methylation and fraction of methylated CGIs of tumor type-specific hyper CGIs for healthy, precursor and tumor mouse model samples. T-ALL samples are marked with a cross. For comparison to the behavior of human tumors, see Fig. 2d. **d** Comparison of the fraction of CpGs methylated at different levels across healthy and tumor samples for mouse models with available healthy tissue. Fractions should only be compared between samples of the same model as deviations due to sequencing technology and sample preparation between different studies are expected

Importantly, we found that the majority of experimentally induced mouse tumors also do not display CGI hypermethylation phenotypes, irrespective of the genetic lesion, environmental stimulant or tissue of origin (Fig. 6a–c, Additional file 2: Fig. S7a-f, Additional file 1: Table S5 and S6). This finding notably expands previous reports on the absence of CGI hypermethylation in select mouse models such as Shh-driven medulloblastoma [51]. Interestingly, this observation also holds for models with genetic mutations of epigenetic regulators that have been associated with widespread CGI hypermethylation in human cancer, such as *Tet2* and *Idh1*, which more recent reports suggest may also be acting to control cell state identity through alternative mechanisms such as enhancer activity or nuclear topology (Fig. 6a–c, Additional file 2: Fig. S7a-f) [52–54].

Notably, although most genetic drivers and experimental conditions did not induce clinical methylation signatures, those associated with T-ALL consistently showed some degree of CGI hypermethylation, including within isolated precursor stages as previously reported [50]. The main factor associated with T-ALL hypermethylation appears to be the tumor type, as mice that develop acute myeloid leukemia (LAML) do not show substantial patient-like CGI methylation even when using the same genetic drivers (e.g. loss of DNMT3A, Fig. 6a and b, Additional file 2: Fig. S7a). However, even for T-ALL, mouse model samples clearly do not reach the same hypermethylation levels as found in patients (Fig. 6a and b).

Our investigation shows that the majority of genetically modified mouse models do not acquire the general features of patient methylomes. However, several studies have previously reported some signs of CGI or promoter methylation using the same or similar data [44, 48, 49, 55, 56]. To examine these disparities more thoroughly, we evaluated the fraction of methylated CGIs (mean methylation > 0.2, introduced in Fig. 2a and d) as we did for the human cellular models above. Using this approach, we do see subtle evidence of epigenetic alterations in some cases, such as the AOM/DSS colon, the C3H liver tumor and the *Flt3-ITD*^*KI*^*/Dnmt3a*^*fl/*+^ LAML model, although these changes remained minor when compared to the effects seen in patients (Additional file 2: Fig. S7a). Similarly, DMR calling for sufficiently sampled models (with more relaxed parameters due to the sparsity of RRBS compared to WGBS) was able to recapitulate some previously reported, generally local hypermethylation events, such as within the PyMT model [44], Additional file 2: Fig. S7g and h). Finally, by inspecting these models at single CpG resolution, we observed some sporadic increases that occur within CGIs (Fig. 6d, Additional file 2:Fig.S8a). In the case of the *Idh1(R132H)*^*KI*^ model, the slight gain of methylation is more global, leading to increases in the genomic background instead of at commonly hypermethylated CGIs in LAML, an effect that could be related to previous reports regarding the role of non-CGI hypermethylation in cis-regulatory elements upon loss of Tet activity [57]. Collectively, none of these more minor changes compare to the overall CGI methylation levels reached in human tumors.

The lack of separation between experimentally induced tumors and controls also holds at the level of hierarchical clustering. For example, pan-cancer hyper or PRC2-target CGI methylation levels primarily separate patient material by healthy versus tumor status, with very few exceptions. In contrast, clustering the different mouse models frequently grouped samples from the same model and tissue together, regardless of their status as healthy or tumor, with the major exception being the comparatively hypermethylated T-ALL tumors that cluster as a clear outgroup (Additional file 2: Fig. S8b). Genome-wide, mouse tumors exhibit mostly stable methylation levels compared to their healthy counterparts, with some hypomethylation visible in specific conditions, particularly for Dnmt3a-deficient LAML or the C3H liver tumor model (Fig. 6d, Additional file 2: Fig. S8a). The relative global stability of these models also appears to contrast with the more common global hypomethylation seen in human patient data (except for acute leukemias, where patients have a stably methylated genome, Additional file 2: Fig. S3e).

Taken together, our results show that the examined genetically (and chemically) engineered mouse models exhibit broadly similar CGI methylation dynamics to those found in the two human culture models. The general absence of CGI hypermethylation might represent a fundamental difference in nuclear regulation, which could have profound implications for mechanistic explorations using these models. The only exception we have found is T-ALL, which partially acquires cancer-like methylation signatures independently of the mutagenic driver and appears to reflect the sensitivity of adaptive immune cells to epigenetic reprogramming in comparison to other cell types. Interestingly, mouse models seem to maintain more stable genomic methylation levels in comparison to the drastic changes we found within transformed human melanocytes and BJ fibroblasts. The differences in DNA methylation dynamics between patients and models also appear to have a limited impact on gene expression, as most genes associated with CGI promoter methylation are regulated by PRC2 and are already silenced prior to transformation (Additional file 2: Fig. S8c-f).

## Discussion

Here we provide extensive data and analyses to support the common notion of CGI hypermethylation in human tumors and highlight the near universal nature of this phenomenon. Specifically, we found that adenocarcinomas, skin cutaneous melanoma and leukemias are generally prone to CGI hypermethylation within PRC2-regulated territories, while sarcomas, thymic and renal cancers are not. Although the exact CGI target profile depends on the tissue of origin and shows variability between patients, cancer-associated CGI methylation is nonetheless distinguishable from almost any healthy control tissue. The degree to which the cancer methylation appears specific to the disease state makes the virtual absence of this pattern in genetically engineered cancer models even more striking. In most cases, even the experimental impairment of demethylation associated pathways (via *Tet2* or *Idh1* mutation) does not induce a drastic change in CGI methylation in mouse models, suggesting that they remain protected from de novo methyltransferase activity in these contexts. This comparable epigenetic stability is in striking contrast to human tumours, including many human premalignant lesions, such as monoclonal B cell lymphocytosis [33], adenoma [32], monoclonal gammopathy of undetermined significance [30, 33], or ductal carcinoma in situ [31], which often already display major departures in CGI regulation.

Given the prevalence of epigenetic dysregulation in patients, it remains unclear why current experimental models appear resistant to similar changes. Previous reports hypothesize that CGI hypermethylation could be reflective of stochastic erosion during unchecked proliferation [58], and tumors in patients have likely grown for substantially longer than the model systems used in these studies. However, this explanation contrasts with the stronger global, presumably proliferation-dependent, hypomethylation observed in the 2D culture models that nonetheless do not methylate tumor-associated CGIs. In these systems, proliferation-associated DNA methylation changes appear to be decoupled from the gain of methylation found at CGIs, suggesting that they must occur at different rates within experimental models compared to patient tumors. As patient cohorts can include outlier samples with relatively low frequencies of CGI hypermethylation, it could be argued that experimental models are correctly approximating the small subset of patient tumors that exhibit minimal DNA methylation changes. However, this narrow interpretation still does not account for the disparity between the majority of patient tumors and the diversity of experimental models examined here, including those that incorporate environmental drivers (AOM/DSS, obesity) or rely on spontaneous downstream mutations (*Apc*^*Min/*+^, C3H). The frequency with which this pattern is found across tumors may indicate a bottleneck or early positive selection for CGI hypermethylation as part of native transformation, a process that would be largely diminished in genetically engineered models designed to accelerate the initial stages of clonal growth [11]. This naturally raises the question of whether CGI hypermethylation is of any ongoing functional necessity within an established human tumor, rather than a pre-determinant of early adaptations. Either way, it remains an important clinical signature with as of yet unclear origins and roles.

Our investigation also highlights possible cell state-specific differences in the likelihood of acquiring a tumor-like methylation landscape. Beyond the handful of intriguing tumor types that appear largely immune to these transitions in patients, the only models that exhibit visibly broad CGI hypermethylation in response to an experimental trigger are mouse models of T-ALL, a unique tumor type with respect to its DNA methylation landscape. T-ALL maintains a globally stable, highly methylated genome (a feature it shares with LAML) [26] that includes the highest number of hypermethylated CGIs and the largest interquartile range of CGI methylation levels within the 450 k array cohort. This unique DNA methylation signature has also been reported in previously published WGBS datasets, where T-ALL samples showed a wide range of CGI methylation levels, from virtually no de novo methylation to complete methylation of thousands of CGIs [26]. The propensity for very high global and CGI-level methylation may be innate to this specific tumor type and linked to its cell of origin, a phenomenon that would be consistent with the unique ability of mouse T-ALL models to acquire some degree of CGI hypermethylation as part of their progression. This cell context-specific interaction is further supported in the data, where the same genetic driver (Dnmt3a KO) is used to direct other tumors (LAML) without equivalent changes in DNA methylation. Whether or not these epigenetic alterations in T-ALL are triggered in the same way or are mechanistically comparable to the majority of solid tumors remains to be seen.

More generally, our results highlight the value of large consortia projects such as TCGA in understanding genetic and epigenetic changes characteristic of tumors, which have already contributed to the development of new therapeutic interventions and diagnostics [59–63]. Our work also shows the importance of performing comparable efforts to systematically evaluate experimental models of tumorigenesis, which have not been undertaken despite their equally important role in understanding tumor biology. In addition to establishing an important contextualization for the current status of the field, our investigation provides a powerful baseline for identifying missing regulatory factors that might drive global changes in DNA methylation. In turn, this will enable the advancement of next-generation cancer models that also incorporate the epigenetic dimension of human tumorigenesis to screen beyond genetic dependencies. It is also worth noting that these principles will likely extend beyond DNA methylation itself, as this modification is tightly linked to other epigenetic layers such as histone modifications and nuclear organization. Ultimately, improved experimental systems would represent an exciting opportunity to explore the causality and role of epigenetic transformation across a wide range of human tumors.

## Conclusions

We establish an analytical benchmark that confirms the striking specificity of CGI hypermethylation across a broad range of human tumors and the clear absence of comparable signatures in purified non-cancerous cell types. We further demonstrate that a diverse number of genetically engineered mouse (*n* = 19) and human (*n* = 2) experimental models do not properly recapitulate similar patterns despite their ability to support unchecked growth. Our results strongly suggest that core principles of epigenetic regulation differ between patient samples and their engineered models. This insight should be of central relevance to the field and provides a valuable lens for the development of improved experimental models that better capture patient biology and enable detailed functional investigation of epigenetic rewiring in tumors.

## Methods

### Human patient samples

#### Melanoma

Patients of the metastatic melanoma study were recruited in the Precision Oncology Program of the Charité Comprehensive Cancer Center (Berlin). Informed consent was obtained from all human subjects included in the study. The study was approved by the local Institutional Review Board of the Charité Universitätsmedizin Berlin (EA4/063/13, Charité Ethics Committee: Charitéplatz 1, 10,117 Berlin, Germany). Eligible patients had a histologically proven metastatic melanoma and exploited all approved therapies. Other inclusion criteria included age ≥ 18 years. We collected fresh metastasis samples for next generation sequencing.

#### Other

Genomic DNA for healthy and primary tumor samples (breast adenocarcinoma, colon adenocarcinoma, kidney renal clear cell carcinoma, hepatocellular carcinoma, lung adenocarcinoma and pancreas adenocarcinoma) was obtained from OriGene. All catalog numbers are listed in Supplementary Table 3. Only tumor samples with a purity ≥ 80% were selected.

### Melanocyte culture and xenografts

Healthy human epidermal melanocytes from ThermoFisher Scientific (Cat. C0025C, donor 1,583,283), and genome-edited versions of these melanocytes, described in Ref. [35], were cultured in hypoxic conditions (37 °C, 5% CO_2_, and 5% O_2_) in M254 media (ThermoFisher, Cat. 5,254,500) supplemented with human melanocyte growth supplement (ThermoFisher, Cat. S0025). Further details on the culture and genome editing strategy are available in Ref. [35]. These melanocytes were not subjected to cell line authentication by STR matching, as a STR reference of these lines does not exist. Mycoplasma testing was conducted during culture and was negative.

For the in vivo xenograft experiments, 1 x 10^6^ cells of one genotype, resuspended in 50 μL of media, were injected intradermally (two injections per mouse, one in each flank) in female NOD.Cg-Prkdc^scid^ Il2rg^tm1Wjl^/SzJ (NSG, stock number: 005557) mice, 4 to 6 weeks old, as previously described in Ref. [35], and under the guidelines and approval of the Massachusetts Institute of Technology Committee for Animal Care (MIT CAC) under protocol 0036–01–15. Xenografts derived from injections of CBTPA cells were grown for approximately 1 month, the ones derived from CBTA and CBTP3 cells were grown for approx. 2 months and the ones derived from CBTP cells were grown for approx. 5 to 6 months. Xenograft samples profiled here do not match the samples presented in Ref. [35], with the exception of two of the CBTP 6 month samples (“xenograft_CBTP_87XL” and “xenograft_CBTP_87XR”), which were present in the publication as “CBTP(6mo) rep1” and “CBTP(6mo) rep2,” respectively. DNA was extracted using the QIAamp DNA Mini Kit (Qiagen) according to the manufacturer’s protocol.

An additional human epidermal melanocyte sample was obtained from Lonza (CC-2586) and short-term cultured in MBM™−4 plus SingleQuots™ of Growth Supplements and Endothelin-3 (Lonza, CC-3249 and CC-4510). Nucleic acid preparations for this sample as well as the melanoma patient samples were performed using the AllPrep DNA/RNA/miRNA Universal Kit (Qiagen, 80,224). DNA concentration was determined on a Qubit Fluorometer.

### Tissue collection of mouse models

#### ***Apc***^***Min/***+^model

These procedures have been performed in a specialized specific-pathogen-free facility, followed all relevant animal welfare guidelines and regulations, and were approved by Harvard University Institutional Animal Care and Use Committee (IACUC) protocol (28–21). Two C57BL6/J-*Apc*^*Min*^ strain mice (Jackson Labs, Cat # 002020) were euthanized at 6 months of age, after the expected emergence of early adenomas within the small intestine. The full intestine was dissected, cleared of waste, and divided into three (Duodenum, Jejunum, Ileum) and two (Cecum and Colon) fractions for the small and large intestine, respectively. Samples were then bisected longitudinally to confirm the presence of macroscopic adenomas in the range of ~ 1–5 mm, after which the tumors were carefully dissected and the position from the cecum and diameter of the tumor were measured. Following isolation of visible benign tumors, the bulk epithelial tissue was purified as described to provide a matched non-cancer tissue control [64]. Both primary “tumors” and isolated epithelia were briefly inspected for purity prior to snap freezing on dry ice. Then, isolated tissue was purified using QIAmp DNA Blood and Tissue Kit with an extended 6-h Proteinase K incubation to improve the bisulfite conversion efficiency.

*Apc*^*fl/*+^ with different *Kras* mutations: The animal procedures were performed under protocol approved by the Beth Israel Deaconess Medical Center IACUC. Mouse colon tumors expressing mutationally activated K-Ras were derived from Fabp1-Cre; *Apc*^*2lox14/*+^; *Kras*^*LSL−G12D/*+^ (or *Kras*^*LSL−G13D/*+^ or *Kras*^*LSL−A146T/*+^) animals [42, 65]. Tumors were isolated during necropsy of moribund animals and immediately snap frozen in liquid nitrogen.

#### KCO model

Animal studies were approved by the Yale University IACUC. For studies of pancreatic mouse tumors, we used the previously generated mouse model harboring *Kras*^*LSL−G12D*^*; Pdx1-Cre (KC)* for pancreas-specific Kras expression [66]. KC mice were crossed with a genetic model of obesity (*Lep*^*Ob/Ob*^) to generate *KC; Lep*^*Ob/*+^ (KCO(het)) and *KC; Lep*^*Ob/Ob*^ (KCO) mice. Mouse genotypes were confirmed by PCR using template tail DNA isolated via HotSHOT DNA extraction and GoTaq Green Mastermix (Promega). Mice were fed standard chow for the duration of the study. At 13 weeks of age, mice were euthanized by CO_2_ asphyxiation and pancreata were rapidly dissected, flash frozen in liquid nitrogen, and stored at − 80C. Tumor burden (% area of pancreas comprised of cancerous lesions) was approximately 10% and 60% for KCO(het) and KCO samples, respectively.

### WGBS library preparation

The DNA was sheared in Covaris micro TUBE AFA Fiber Pre-Slit Snap-Cap tubes (SKU: 520,045) and cleaned up with the Zymo DNA Clean & Concentrator-5 kit (#D4013) following manufacturer’s guidelines. Sheared gDNA was bisulfite converted following the manufacturer’s guidelines with the EZ DNA Methylation-Gold Kit (Zymo #D5005), and libraries were prepared using the Accel-NGS Methyl-seq DNA library kit (Swift Biosciences, #30,024-SWI) or the xGen Methyl-Seq DNA Library Prep Kit, (IDT, #10,009,824). Libraries were cleaned using Agencourt AMPure XP beads (Beckman Coulter, #A63881) and the final libraries were sequenced on a NovaSeq platform (Illumina) yielding 150 bp paired-end reads.

### RRBS library preparation

Concentration of genomic DNA (gDNA) was quantified using a Qubit 3.0 Fluorometer. RRBS [67, 68] was performed on 100 ng gDNA of each sample using the Swift Biosciences ACCEL-NGS Methyl-Seq DNA Library Kit (#30,024) or NuGen Ovation RRBS Methyl-Seq System (Tecan, #0353) following the manufacturer’s recommendations with the following modifications: For the *Apc*^*Min/*+^ samples, the DNA was fragmented using Covaris S2 and purified with Agencourt RNAclean XP beads (Beckman Coulter, #A63987) (1.8X). For all samples, after the final repair step, the bisulfite conversion of DNA was conducted using the Qiagen EpiTect Fast Bisulfite Conversion kit (Qiagen, #59,824) following the manufacturer’s recommendations, eluting the bisulfite converted DNA in 22–23 µl EB. Libraries were amplified with 10–12 cycles of PCR. Amplified library purification with Agencourt RNAclean XP beads (Beckman Coulter, #A63987) was performed twice (1–1.2X). The purified libraries were sequenced on a NextSeq 500 or NovaSeq 6000 platform (Illumina, 76 bp single-end reads).

### Target enrichment library preparation

Genomic DNA (gDNA) was purified using the DNeasy Blood & Tissue Kit (Qiagen, #69,504) following the manufacturer’s instructions. Snap-frozen tissues were cut into small pieces (~ 25 mg) and digested overnight in Buffer ATL with Proteinase K and collected using the DNeasy Mini spin column. WGBS libraries were then prepared as above using the same kit and protocol. For the KCO samples, we performed target enrichment of previously described PRC2-targeted CGIs [69] using 200 ng of each WGBS library from two KCO(het) and five KCO mice, pooled and mixed with a Twist Custom Methylation Panel specific for targeted CGIs, Universal Blockers (Twist, #100,578), and Methylation Enhancer (Twist, #103,557), then dried using a SpeedVac system. The dried product was resuspended with fast hybridization mix and enhancer (Twist, #100,930), and hybridized at 60 °C for 2 h. Targeted sequences were captured and purified using Streptavidin Binding beads (Twist, #100,983), and the final library pool was amplified using 2 × Equinox Library Amplification Mix (Twist, #104,108) with 11 PCR cycles based on the total length of targeted regions. The final library was purified with DNA Purification Beads (Twist, #100,983) and sequenced on a NovaSeq platform (150 bp paired-end reads).

### WGBS and target enrichment processing

#### This study

Raw reads were trimmed (adapter and quality) using cutadapt (version 4.1; parameters: --quality-cutoff 20 --overlap 5 --minimum-length 25; Illumina TruSeq adapter clipped from both reads), followed by trimming of 10 and 5 nucleotides from the 5’ and 3’ end of the first read and 15 and 5 nucleotides from the 5’ and 3’ end of the second read [70]. The trimmed reads were aligned to the human (hg19) or mouse (mm10) genome using BSMAP (version 2.90; parameters: -v 0.1 -s 16 -q 20 -w 100 -S 1 -u -R) [71]. Xenograft samples were aligned to a combined hg19 and mm10 genome. Duplicates were removed using the ‘MarkDuplicates’ command from GATK (version 4.2.5.0; --VALIDATION_STRINGENCY = LENIENT --REMOVE_DUPLICATES = true) [72]. Methylation rates were called using the MOABS package with the ‘mcall’ function (version 1.3.9.6; default parameters) [73]. For the xenograft samples, only hg19 CpGs were considered. All analyses were restricted to autosomes and only CpGs covered by at least 10 reads (for WGBS also at most 150 reads) were considered for downstream analyses.

#### Publicly available data

For the WGBS data sets of the AOM/DSS model, replicates were merged and analyses were restricted to CpGs with at least five and at most 150 reads to increase the overall lower coverage (in line with the mouse model RRBS datasets these samples were compared to that were also filtered for a minimum of five reads). For publicly available human WGBS data sets (LAML, T-ALL, sorted cell types), the already processed data was obtained from the respective sources (see data availability statement) and coverage was filtered subsequently as described above for the other human WGBS samples (LAML samples were lifted to hg19 before).

### RRBS processing

#### This study

Raw reads were trimmed (adapter and quality) using cutadapt (version 4.1; parameters: –quality-cutoff 20 –overlap 5 –minimum-length 25; Illumina TruSeq adapter), followed by NuGEN diversity adapter trimming (https://github.com/nugentechnologies/NuMetRRBS). The trimmed reads were aligned to the mouse genome (mm10) using BSMAP (version 2.90; parameters: -v 0.1 -s 12 -q 20 -w 100 -S 1 -u -R -D C-CGG). Aligned reads were deduplicated based on unique molecular identifiers (UMIs) using NuDup (https://github.com/nugentechnologies/nudup; parameters: –start 6 –length 6). Methylation rates were called the MOABS package with the “mcall” function (version 1.3.9.6; default parameters). All analyses were restricted to autosomes and only CpGs covered by at least five and at maximum 150 reads were considered for downstream analyses.

#### Publicly available data

The same steps were performed with the exception of NuGEN diversity adapter trimming and NuDup deduplication as these libraries were not generated with diversity adapter or UMIs.

### 450 k array processing

Publicly available acute lymphoblastic leukemia (ALL) data sets and BJ samples from the senescence/transformation model generated using the Illumina Infinium HumanMethylation450 BeadChip [39, 74] were processed using the Minfi R package following similar processing steps as used within TCGA (version 1.32.0) [75]. Data was loaded with the “read.metharray.exp” function. Failed positions were identified using the function “detectionP” (parameters: type = “m + u”). Data was normalized using the Noob normalization (“preprocessNoob,” parameters: dyeMethod = “single”). The methylation ratio was computed using the “ratioConvert” function (parameters: what = “both,” keepCN = TRUE). Probes overlapping SNPs (“addSnpInfo,” “dropLociWithSnp,” parameters: snps = c(“Probe,” “SBE,” “CpG”), maf = 0.01) or non-CpG positions, located on the sex chromosomes, that failed the detection test (*p*-value > 0.05) or frequently cross-react [76] were excluded from the analyses. Additionally, processed beta values from 26 different tumor types with available healthy tissue were downloaded from TCGA (a CD34 + healthy sample from the ALL cohort was used as control for LAML) and positions were reduced to the probes passing all filtering steps during the processing of the ALL samples. Consensus purity estimates for TCGA tumor samples were obtained from Aran et al. [77].

### RNA-seq processing

Raw reads of publicly available mouse model expression data were trimmed (adapter and quality) with cutadapt (version 4.1; parameters: --quality-cutoff 20 --overlap 5 --minimum-length 25 --interleaved --adapter AGATCGGAAGAGC -A AGATCGGAAGAGC), followed by poly-A trimming with cutadapt (parameters: --interleaved --overlap 20 --minimum-length --adapter “A[100]” --adapter “T[100]”). Reads were aligned to the mouse reference genome (mm10) using STAR (version 2.7.11b; parameters: --runMode alignReads --chimSegmentMin 20 --outSAMstrandField intronMotif --quantMode GeneCounts) [78] and transcripts were quantified using stringtie (version 2.0.6; parameters: -e) [79] with the GENCODE annotation (release VM19).

### Feature annotation

One-kilobase genomic tiles were generated by segmenting the genome using bedtools makewindows (version 2.30.0; parameters: -w 1000 -s 1000) [80]. Location of solo-WCGW CpGs in common (defined across a pan-cancer cohort) partially methylated domains on the 450 k array for hg19 were downloaded from https://zwdzwd.github.io/pmd [9]. The mm10 and hg19 gene annotations were downloaded from GENCODE (V19, VM19). Promoters were defined as 1500 bp upstream and 500 bp downstream of the transcription start site (TSS). ChromHMM annotations for the human embryonic stem cell (hESC) line HUES64 and penis foreskin melanocytes were downloaded from Roadmap (E016, E061).

Annotations of CpG islands (CGIs) for hg19 and mm10 were downloaded from UCSC. CGI shores were defined as the 2 kb flanking each side of a CGI while CGI shelves were defined as the outer 2 kb flanking each shore. The remaining parts of the genome were declared as “open water.” For both species, CGIs were defined as promoter CGI if at least 20% of the CGI or the promoter overlapped. The chromatin state of hg19 CGIs was defined as the ChromHMM state with the largest overlap with each island. A CGI was defined to be targeted by PRC2 in hESCs if the corresponding chromatin state was one of 10_TssBiv, 11_BivFlnk, 12_EnhBiv, 13_ReprPC and 14_ReprPCWk. For mm10, CGIs were defined to be targeted by PRC2 in mouse ESCs if at least 20% of the CGI overlapped with a H3K27me3 domain as described previously [81]. For downstream analyses, PRC2 target sets were reduced to CGIs within gene promoters.

### PMD calling

PMDs of the wild type melanocyte sample were called using DNMTools with the “pmd” function (version 1.4.2, default parameters) [82]. Close PMD calls were merged using bedtools mergeBed (version 2.30.0; parameters: -d 200,000) to avoid the classification of, e.g., a single gene body as HMD. PMDs were filtered by minimum size (> 200 kb). HMDs were then defined as the complement of PMDs excluding regions > 100 kb that were not covered by the samples (such as centromeres). Subsequently, 100-kb tiles from a hg19 segmentation were classified as HMD or PMD depending on the largest overlap per tile.

### Feature-level DNA methylation analysis

All analyses were performed using R-4.2.2 if not noted otherwise. In almost all cases, healthy and tumor samples that were compared with each other were obtained from the same sources, which decreases the impact of potential batch effects across studies.

For WGBS and RRBS samples, the arithmetic mean was calculated across features (tiles, CGIs). A feature was only considered if at least three CpGs were covered within a region. For the publicly available sorted cell type data sets, samples were subsequently also averaged per cell type for each CGI. For this, the refined group defined by Loyfer et al. (Ref. [27]) was chosen and as an additional refinement, memory B and T cells were assigned to their own groups separately from other B or T cells due to known differences in CGI methylation levels over the course of maturation [28, 29]. For samples profiled with the 450 k array, the average methylation of each CGI was computed using beta values of probes located within the respective CGI. These measurements were then further summarized to calculate the median per condition, tumor type, and/or tumor stage or the fraction of methylated CGIs. A CGI was considered methylated with a mean methylation > 0.2.

Both the median and the fraction of methylated CGIs were used as metrics to assess hypermethylation of different CGI sets (see next section). The median level of a CGI set provides a robust summary of CGI hypermethylation levels per sample. However, it might also fail to show slight elevations or corrosion events in samples with overall lower CGI methylation. For this purpose, we employed the fraction of methylated CGIs as an additional metric that shows a greater dynamic range and is thus susceptible to more subtle changes.

In comparison to WGBS, the 450 k array spans a limited number of CpGs that are enriched for regulatory features such as CGIs, promoters, enhancers, and gene bodies [83]. This represents a specific challenge for estimating global background methylation levels independent of regulatory function. A study by Zhou et al. found that specific isolated CpGs in a WCGW context (termed solo-WCGW CpGs) in partially methylated domains are most prone to hypomethylation and therefore provide an accurate measurement to determine the degree of hypomethylation in a sample [9]. 4832 of these CpGs are covered by the 450 k array after the applied filtering steps as described above and located within common (defined across a pan-cancer cohort) PMDs. Therefore, these probes were used in order to compare global DNA methylation dynamics of 450 k array samples.

Genome browser tracks were generated using IGV (version 2.15.2) [84].

### Definition of hypermethylated CGI sets

Hypermethylated (hyper) CGIs per tumor sample were defined as follows: For each CGI, first the median signature of the respective healthy control samples was defined (median over the average methylation of each healthy sample per CGI). Then the average methylation of each CGI in the malignant sample was compared to the normal signature. CGIs that were unmethylated in the healthy condition (methylation ≤ 0.2), methylated in the malignant sample (methylation > 0.2) and with a difference > 0.1 between malignant and median normal signature were termed hyper CGIs for the respective sample.

Tumor type-specific hypermethylated CGIs were defined based on the sets of hyper CGIs defined for each tumor sample (see above): First, CGIs that were termed hypermethylated in at least 50% of tumor samples of a specific type were considered as “common” hyper CGIs for that type referred to as “hyper tumor type” in the text and figures. CGIs selected as tumor type-specific hyper CGIs for at least eight (30%) of all considered tumor types were termed "commonly hypermethylated pan-cancer".

For the analysis of hyper CGIs with different control samples, the procedure defined above was repeated with each normal signature (associated with the 26 tumor types) as control for each tumor type. Then the fraction of CGIs recovered from (or found in addition to) the original set with the matching control tissue was defined based on the overlap (or set difference) of each hyper CGI set with the original set.

In order to define hyper CGI sets in the mouse genome, similar/conserved CGIs between mouse and human were identified by lifting mm10 CGIs to hg19 using UCSCtools “liftOver.” The resulting coordinates were then overlapped with the actual human CGIs (if multiple human CGIs showed an overlap, all of them were considered). Then the subset of mouse CGIs for which a human hyper CGI was found was used for the respective hyper CGI set.

Heatmaps of CGI sets and WGBS or RRBS samples were generated using the R package ComplexHeatmap (version 2.14.0) [85].

### Hyper CGI saturation analysis

In order to assess how uniformly the sets of common hyper CGIs are sampled across the tumor samples of each type, a saturation analysis was performed (Fig. 1e). For this purpose, only tumor types with at least 100 samples were considered. For each tumor type, 100 iterations were performed and within each iteration, 100 tumors were randomly sampled, followed by a calculation of the cumulative proportion of hyperCGIs added with each subsequent sample. The results were then averaged across the 100 iterations in order to provide a more stable assessment of the degree to which individual samples contribute additional information to each tumor type-specific hyper CGI profile.

### DMR calling

DMRs were called using metilene (version 0.2–8; parameters WGBS: -m 10 -d 0.2 -c 1 -f 1 -M 300 -v 0.7; parameters RRBS: -m 5 -d 0.2 -c 1 -f 1 -M 300 -v 0.7) and filtered by *q* value < 0.05 [86]. DMRs were assigned to CGI-related features or chromatin states based on the maximum overlap between them. The chromatin states as annotated by ChromHMM for penis foreskin melanocytes were grouped the following way: Active TSS (1_TssA, 2_TssAFlnk), Bivalent TSS (10_TssBiv, 11_BivFlnk), Transcript (3_TxFlnk, 4_Tx, 5_TxWk), Enhancer (6_EnhG, 7_Enh), Bivalent enhancer (12_EnhBiv), Heterochromatin (8_ZNF/Rpts, 9_Het), Polycomb-repressed (13_ReprPC, 14_ReprPCWk) and Quiescent (15_Quies). Random background DMRs were calculated as described previously [87]. The enrichment of DMRs against background DMRs of a specific feature was calculated as the log2-transformed ratio of the fractions of DMRs and background DMRs within that feature. The overlap between different DMR sets was calculated by first creating a list of merged regions of all considered sets and then calculating the overlap between each DMR set and the merged regions. Heatmaps were again generated using ComplexHeatmap.

### CpG-level DNA methylation analysis

For the CpG-level analysis in mouse models, CpGs in the genomic background (outside CGIs and the flanking 4 kb on each side of a CGI) and within hyper CGIs of the respective tumor types were grouped into methylation levels between 0 and 1 (0.1 steps). The shift in the CpG methylation distribution between tumor and healthy samples was calculated by first averaging the fraction of each level across healthy samples for each model and then subtracting it from the respective fraction of each tumor sample.

### Overrepresentation analysis

Overrepresentation analysis of genes with promoters overlapping either hyper CGI or DMR sets was performed using the R package (and function) WebGestaltR (version 0.4.6; parameters: minNum = 10, maxNum = 500, sigMethod = “top,” topThr = 10, enrichMethod = “ORA,” enrichDatabase = “geneontology_Biological_Process,” referenceSet = “genome”) [88].

### Mitotic age scores and methylation clock

The R package (and function) boostme was used to impute missing values in the WGBS data sets of the melanocyte model and melanoma patients (version: 0.1.0; parameters: minCov = 5, sampleAvg = FALSE, threads = 10) [89]. Scores estimating mitotic history (epiTOC1) [90], stemTOC [91], epiCMIT hyper/hypo [92], HypoClock [93]) were then calculated based on the resulting, imputed data sets using the R package (and function) EpiMitClocks (version: 0.1.0; default parameters) [91]. Similarly, Horvath’s methylation clock [94] was applied using the R package methylclock with the “DNAmAge” function (version: 1.4.0; default parameters) [95].

## Peer review information

Wenjing She was the primary editor of this article and managed its editorial process and peer review in collaboration with the rest of the editorial team. The peer-review history is available in the online version of this article.

## Supplementary Information

Additional file 1. Table S1: Overview and DNA methylation summary of the pan-cancer 450 k array cohort including healthy, tumor and metastasis samples. Table S2: Coordinates of PRC2 target and hyper CGIs(hg19). Table S3: Sequencing statistics and DNA methylation summary of pan-cancer healthy, tumor and melanocyte model WGBS samples. Table S4: Overview and DNA methylation summary of the in vitro 450 k array cohort including senescent and transformed BJ samples. Table S5: Sequencing statistics and DNA methylation summary of the mouse model cohort. Table S6: Coordinates of PRC2 target and hyper CGIs (mm10).

Additional file 2. Fig. S1: Characterization of different hyper CGI sets. Fig. S2: Methylated CGIs in healthy cell types Fig. S3: CGI hypermethylation metrics across different assays. Fig. S4: Global pan-cancer DNA methylation dynamics of melanoma models and patients. Fig. S5: Differentially methylated regions of patients or melanocyte models compared to control samples. Fig. S6: Global methylation depletion in senescence and transformation model. Fig. S7: Differentially methylated regions between tumor and healthy tissue in mouse models. Fig. S8: Transcriptional state of genes associated with hyper CGIs.

## Data Availability

Raw data of the melanocyte model and human patient samples have been deposited at the European Genome-phenome Archive under the accession number EGAS50000000902 [96]. *Apc*^*Min/+*^, *Apc*^*fl/+*^/*Kras*^*G12D/+*^, *Apc*^*fl/+*^/*Kras*^*G13D/+*^, *Apc*^*fl/+*^/*Kras*^*A146T/+*^ and KCO model sequencing and corresponding processed data have been deposited in the Gene Expression Omnibus and are accessible under GSE244634 [97]. Processed data of the human samples together with source data and code used in this study have been deposited at Zenodo [98]. A script that allows the visualization of new cancer models and/or primary tumor data together with the reference sequencing data from this study has been deposited at GitHub under the BSD-3-Clause license (https://github.com/sarahet/CancerModelDNAme) [99]. Publicly available 450k array data sets have been obtained from TCGA, GSE91069 [39, 100] and GSE49031 [74, 101]. Processed WGBS methylation calls of hematopoietic multipotent progenitor cells and LAML samples were downloaded from the Blueprint epigenome project (http://dcc.blueprint-epigenome.eu/). Similarly, precursor T cells and T-ALL WGBS samples were obtained from EGAS00001005203 [26, 102] and sorted healthy human cell types from GSE186458 [27, 103]. Raw data of previously published mouse model samples have been obtained from GSE83623 [44, 104], GSE57569 [105, 106], GSE111420 [55, 107], GSE67392 [45, 108], GSE60264 [47, 109], GSE61971 [46, 110], GSE57114 [49, 111], GSE38687 [48, 112] and GSE155339 [50, 113]. Gene expression data has been obtained from TCGA via the Xena Functional Genomics Explorer in TPM format (https://xenabrowser.net) and as raw data from GSE76772 [114, 115] and GSE61969 [46, 116]. Processed data from available single-cell RNA-seq of the human melanoma model has been obtained from the Single Cell Portal [35]. The results shown here are in part based upon data generated by the TCGA Research Network: https://www.cancer.gov/tcga. This study makes use of data generated by the Blueprint Consortium. A full list of the investigators who contributed to the generation of the data is available from www.blueprint-epigenome.eu. Funding for the project was provided by the European Union's Seventh Framework Programme (FP7/2007-2013) under grant agreement no 282510 BLUEPRINT. This study makes use of data generated by the St. Jude Children’s Research Hospital – Washington University Pediatric Cancer Genome Project [26].
