# Supplementary material for: Direct genetic transformation bypasses tumor-associated DNA methylation alterations
Source: Genome Biol. 2025 Jul 17;26:212. doi: 10.1186/s13059-025-03650-2 (PMC12273271; doi:10.1186/s13059-025-03650-2)
Supplement: Supplementary file 2 — Additional file 2. Fig. S1: Characterization of different hyper CGI sets. Fig. S2: Methylated CGIs in healthy cell types Fig. S3: CGI hypermethylation metrics across different assays. Fig. S4: Global pan-cancer DNA methylation dynamics of melanoma models and patients. Fig. S5: Differentially methylated regions of patients or melanocyte models compared to control samples. Fig. S6: Global methylation depletion in senescence and transformation model. Fig. S7: Differentially methylated regions between tumor and healthy tissue in mouse models. Fig. S8: Transcriptional state of genes associated with hyper CGIs. [file 13059_2025_3650_MOESM2_ESM.docx]

**Supplementary Figures and Legends**


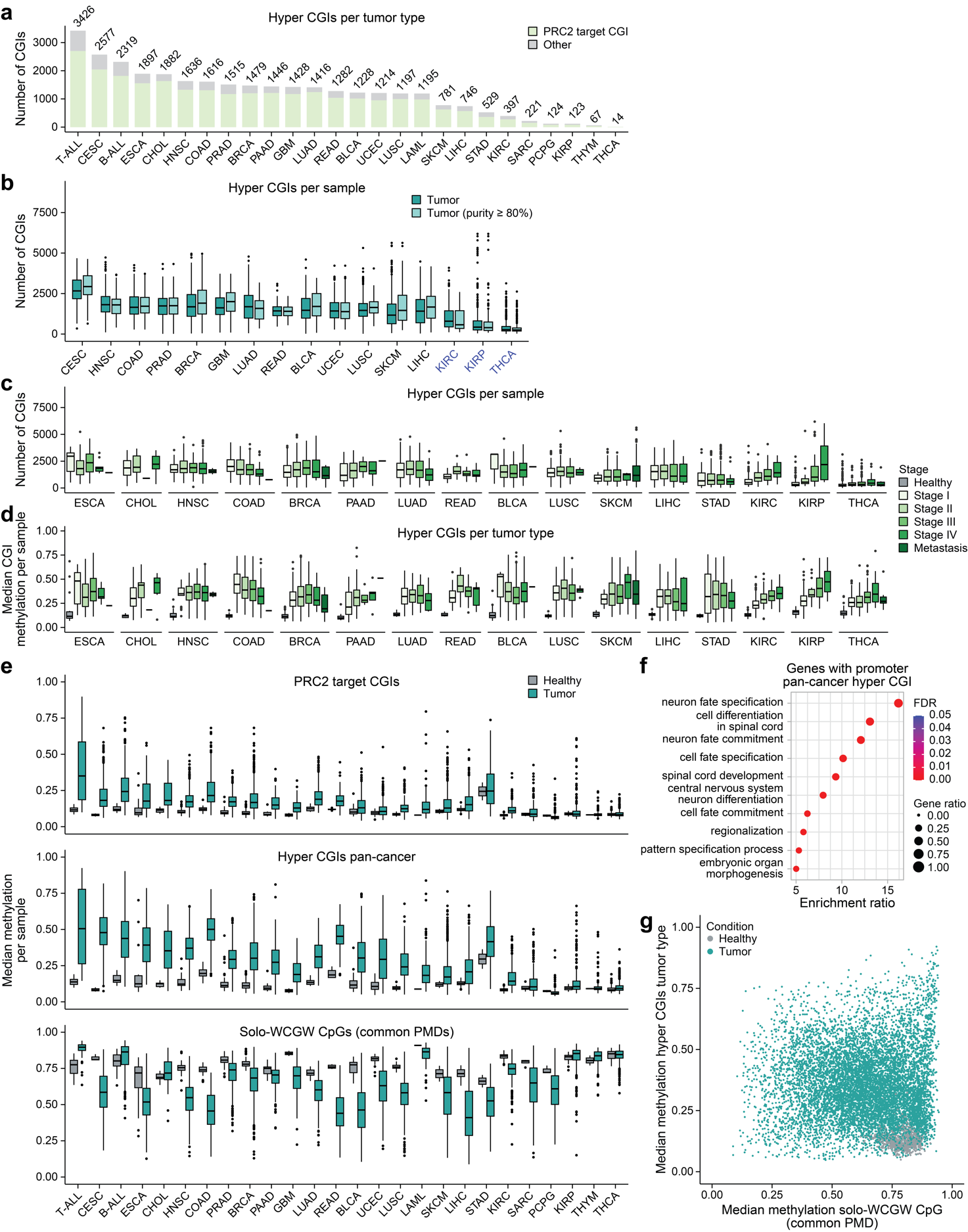


**Fig. S1: Characterization of different hyper CGI sets**

**a)** Barplot indicating the fraction of tumor type-specific hyper CGI sets that is regulated by PRC2 in human embryonic stem cells.

**b)** Number of hyper CGIs per tumor sample per type shown for all patients and separately those with an estimated tumor purity ≥ 80%. Only tumor types with available purity estimation were selected (Aran et al. Nat Comms 2015). Lines denote the median, edges denote the IQR, whiskers denote 1.5 × IQR and minima/maxima are represented by dots.

**c)** Number of hyper CGIs per tumor sample per type split by tumor stage. Only tumor types with available stage information are displayed. For kidney cancer, only later stages seem to be prone to CGI hypermethylation, which are underrepresented in this cohort (41% stage III/IV in KIRC and 26% stage III/IV in KIRP). Lines denote the median, edges denote the IQR, whiskers denote 1.5 × IQR and minima/maxima are represented by dots.

**d)** Boxplot showing the median methylation across each common tumor type-specific hyper CGI set for healthy and tumor samples, split by clinical stage. Lines denote the median, edges denote the IQR, whiskers denote 1.5 × IQR and minima/maxima are represented by dots.

**e)** Boxplot showing the median methylation for PRC2 target CGIs, pan-cancer hyper CGIs and solo-WCGW CpGs in common partially methylated domains (PMDs, approximating global methylation levels on the 450k array, Zhou et al. Nature Genetics 2018) for healthy and primary tumor samples of 26 tumor types. Lines denote the median, edges IQR, whiskers denote 1.5 × IQR and minima/maxima are represented by dots.

**f)** Overrepresentation analysis of genes whose promoters overlap pan-cancer hyper CGIs (biological processes). Genes are enriched in neural and broader developmental functions, consistent with regulation by PRC2.

**g)** Scatterplot showing the relation between median hyper CGI (per tumor type) and solo-WCGW CpG methylation levels for healthy and tumor samples. Healthy samples exhibit a somatic bimodal methylation landscape characterized by low CGI and high genome-wide methylation levels, while tumors show varying degrees of CGI hyper- and/or global hypomethylation.


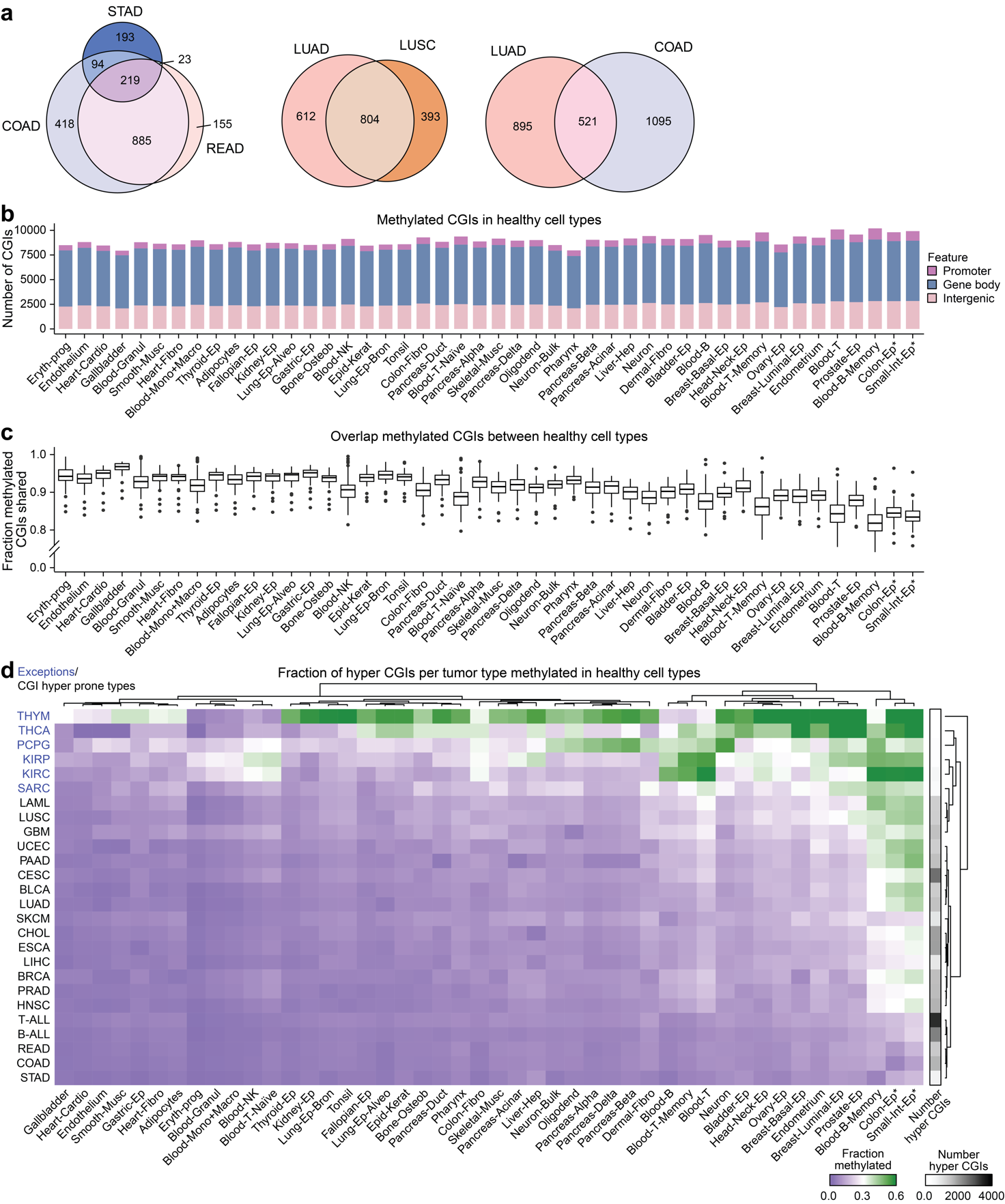


**Fig. S2: Methylated CGIs in healthy cell types**

**a)** Overlap of hyper CGIs between different tumor types originating from related (within digestive tract, within lung) or more distinct tissues (between colon and lung). The overlap of hyper CGIs between lung adenocarcinoma (LUAD) and colon adenocarcinoma (COAD) is smaller than the overlap between lung adeno- and squamous cell carcinoma (LUSC) or stomach (STAD), rectal (READ) and colon adenocarcinoma.

**b)** Barplot showing the number of methylated CGIs (methylation > 0.2) for healthy tissues. The proportion of CGIs in promoters, gene bodies and intergenic regions is indicated.

**c)** Boxplot showing the overlap of methylated CGIs between different cell types as a fraction of each cell type’s specific set when compared to any other cell type. Lines denote the median, edges denote the IQR, whiskers denote 1.5 × IQR and minima/maxima are represented by dots.

**d)** Heatmap showing the fraction of hyper CGIs per tumor type that are methylated across a large cohort of healthy cell types. With the exception of six tumor types with low hyper CGI numbers, the fraction of overlapping CGIs is low (memory B cells as well as the colon and small intestine epithelium methylate larger numbers of tumor-associated CGIs for potential reasons explained in text).


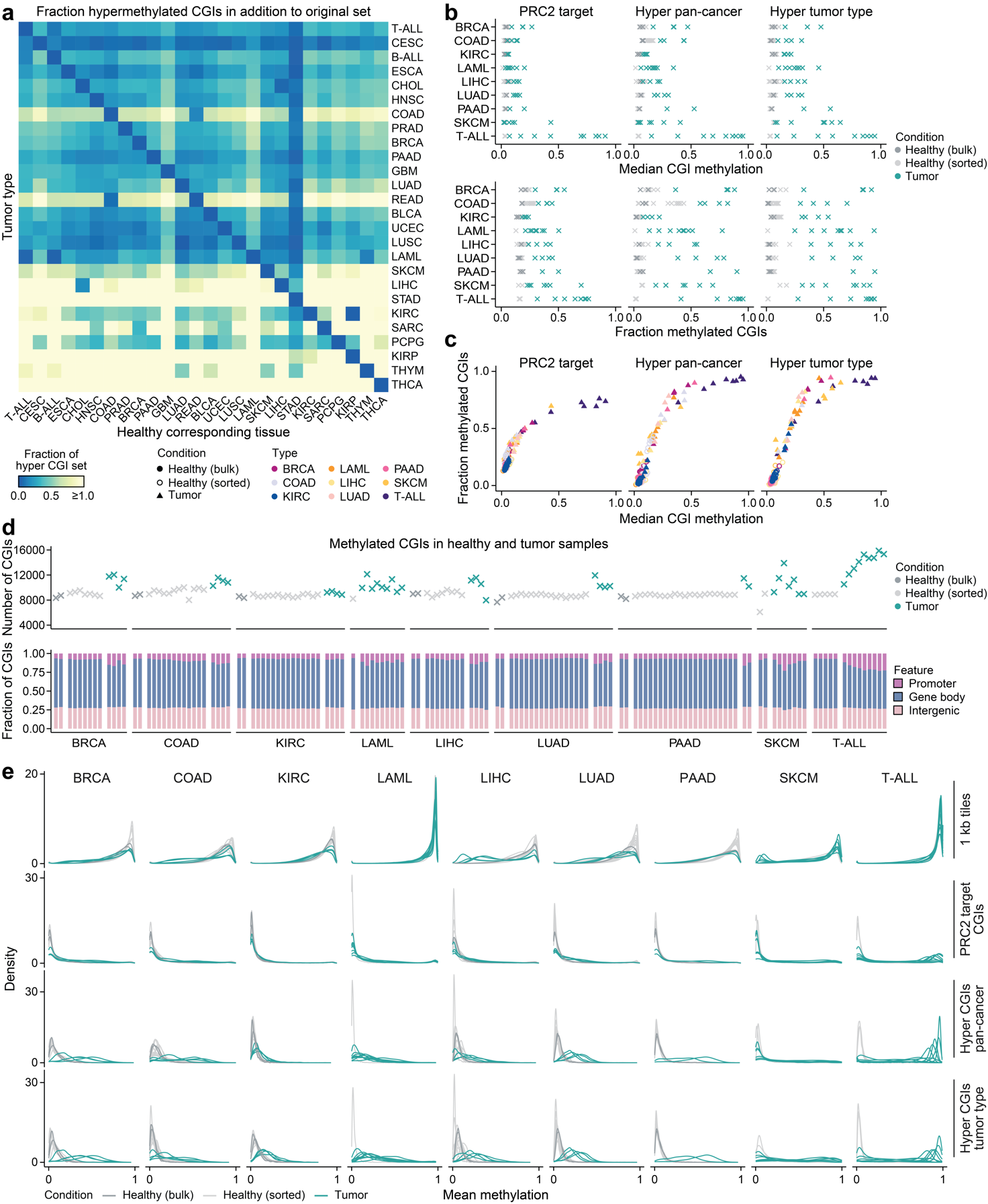


**Fig. S3: CGI hypermethylation metrics across different assays**

**a)** Heatmap displaying the frequency of false positive hyper CGIs that are called when using any available healthy tissue as a prospective control. The frequency of false positives is indicated as a fraction of the original set (as called using the proper control) to provide a relation to its size. The diagonal is always 0, as no additional CGIs are detected when using the correct control tissue.

**b)** Median methylation (top) and fraction of methylated CGIs (bottom) for normal and tumor WGBS samples, including for the set of PRC2 target CGIs, pan-cancer hyper CGIs and tumor type-specific hyper CGIs.

**c)** Scatterplot showing the median methylation and fraction of methylated CGIs for normal and tumor WGBS samples, including for the set of PRC2 target CGIs, pan-cancer hyper CGIs and tumor type-specific hyper CGI. Same panel as in **Fig. 2d** but colored by tumor type with condition indicated by the shape.

**d)** Top: Number of methylated CGIs within healthy and tumor samples. Bottom: Distribution of methylated CGIs according to gene-related features. Tumor samples have more methylated CGIs and simultaneously increase the number of methylated CGIs found in promoters.

**e)** Density plot of one kb tiles and different CGI sets for all WGBS patient samples (healthy and tumor). Tumors show varying degrees of global hypo- and CGI hypermethylation.


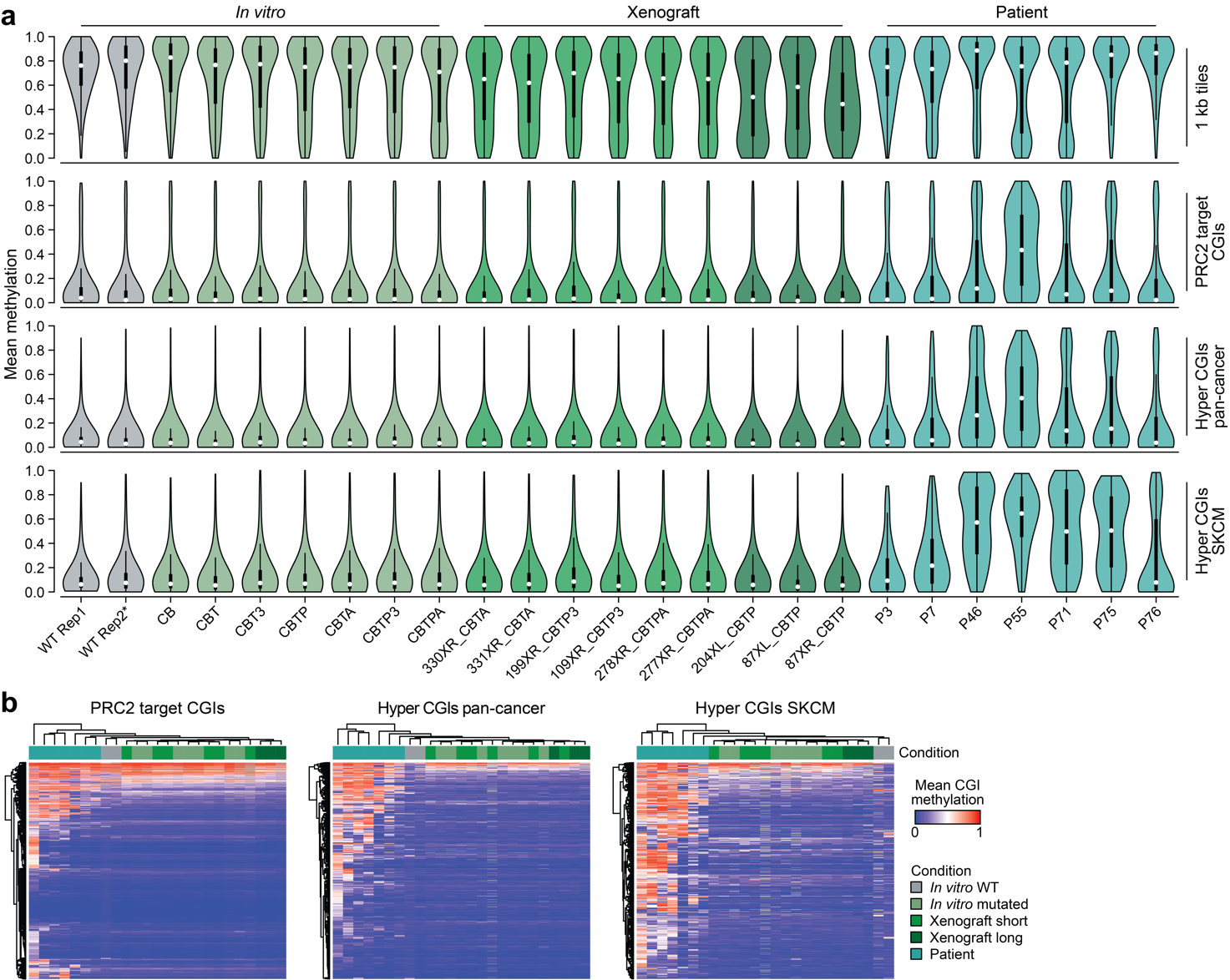


**Fig. S4: Global DNA methylation dynamics of melanoma models and patients**

**a)** Violin plot of one kb tiles and different CGI sets for all WGBS samples of the melanocyte model and melanoma patients. Lines denote the median, edges denote the IQR, whiskers denote 1.5 × IQR and minima/maxima are indicated by the violin plot range (* indicates the wild type replicate that was used to subsequently introduce mutations).

**b)** Heatmaps and hierarchical clustering of melanoma patients and melanocyte model samples based on different CGI sets. Patients consistently cluster separately from the model due to the difference in CGI methylation levels (hypermethylated in patients but not in the engineered melanocytes and resulting xenografts).


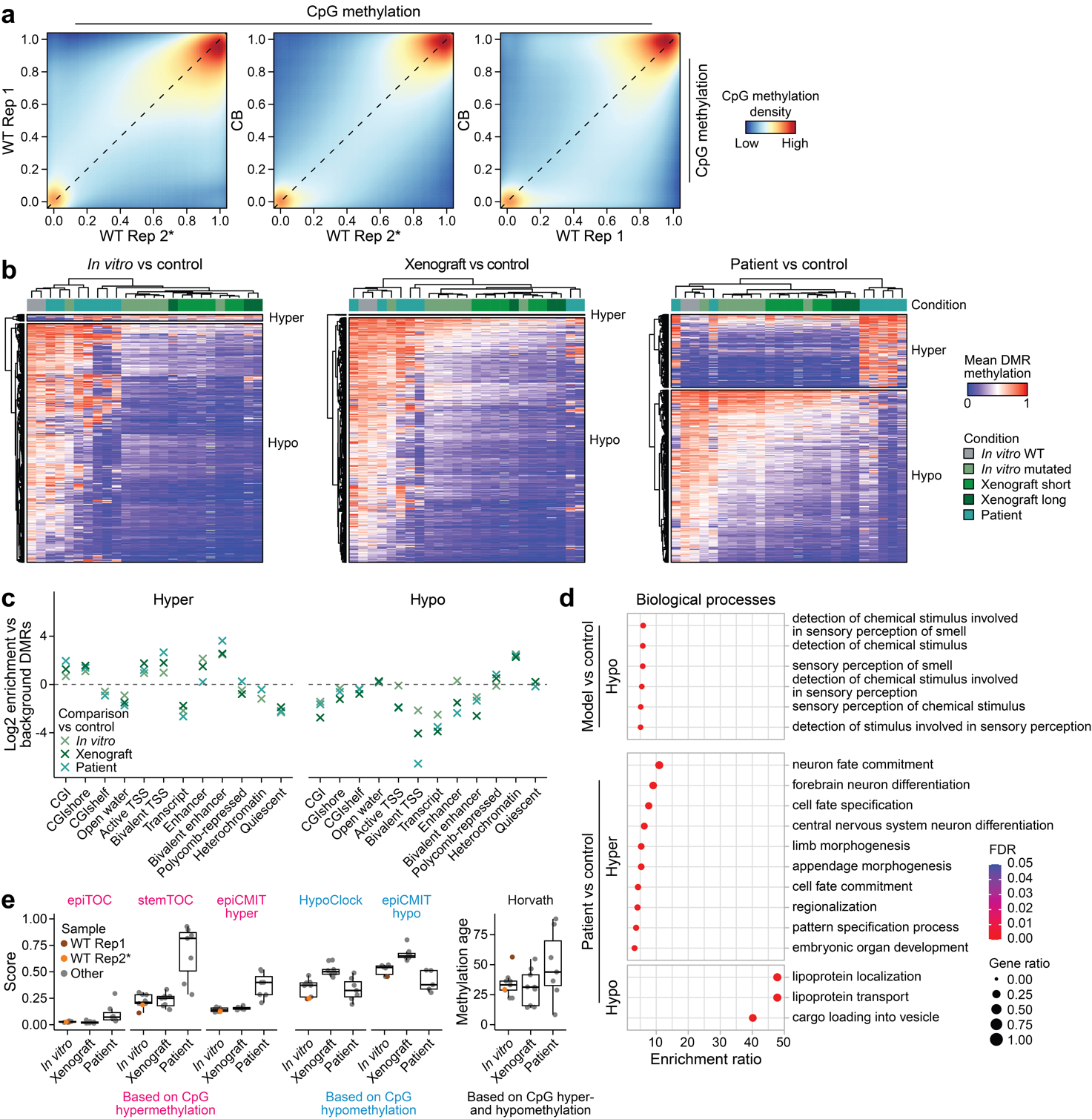


**Fig. S5: Differentially methylated regions of patients or melanocyte models compared to control samples**

**a)** CpG-wise density plot comparing the two wild type melanocyte replicates with the first mutated sample (CB, * indicates the matched wild type replicate that was used to introduce experimental mutations).

**b)** Heatmap and hierarchical clustering of wild type and mutated melanocyte samples (including xenografts) as well as patients based on three different DMR sets.

**c)** Log2 enrichment of DMRs in genomic features and chromatin states compared to a set of randomly sampled background DMRs with similar properties (see Methods).

**d)** Overrepresentation analysis of genes with promoters overlapping different DMR sets (biological processes, the two model DMR sets have been merged for this analysis). No significant enrichments were detected for hypermethylated DMRs in the melanocyte model compared to the control.

**e)** Metrics estimating mitotic age from CpGs tracking accumulated hyper- or hypomethylation (left) and Horvath’s methylation clock estimating methylation age (right, based on slightly more hyper- than hypomethylation-prone CpGs). Depending on the score type, either patients or xenografts have longer predicted mitotic histories due to the strong differences in CGI hyper- and PMD hypomethylation between tumors and models (* indicates the wild type replicate that was used to subsequently introduce mutations).


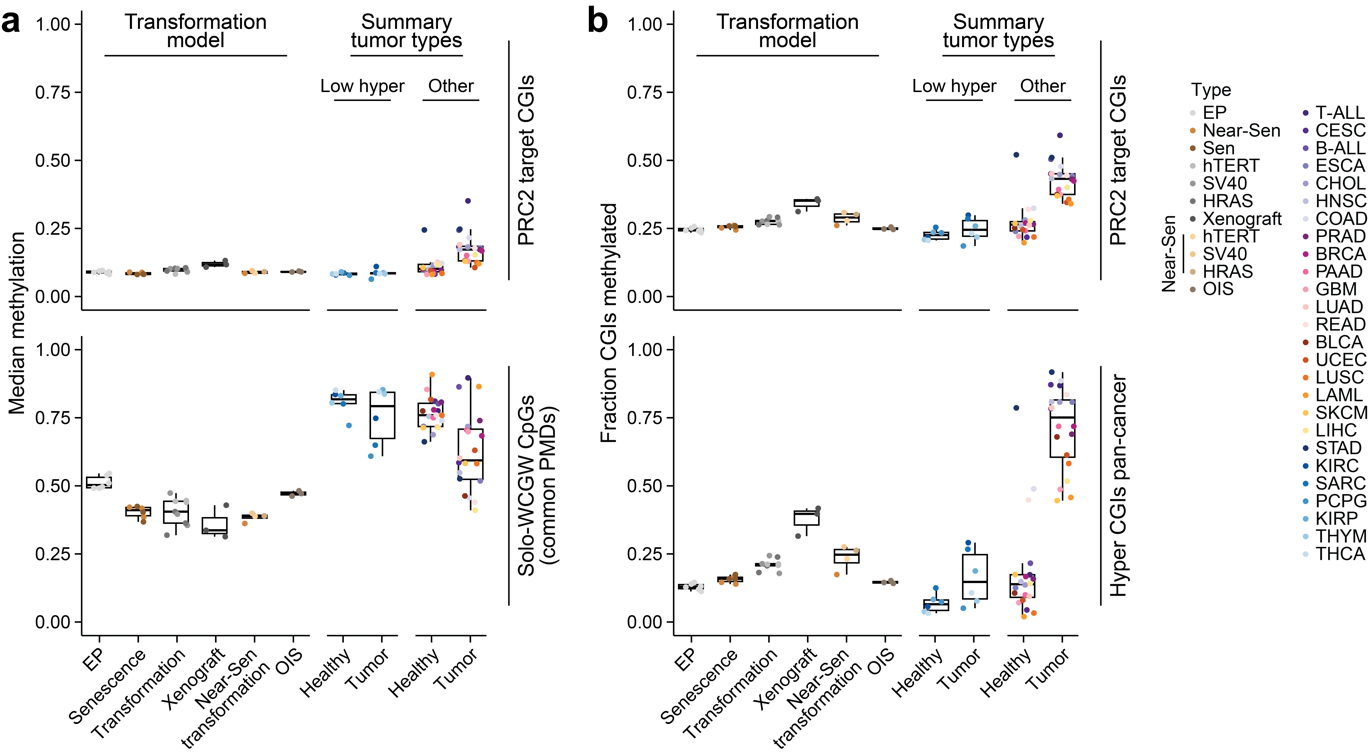


**Fig. S6: Global methylation depletion in senescence and transformation model**

**a)** Boxplot showing the median methylation for PRC2 target CGIs and solo-WCGW CpGs in common PMDs for samples of the BJ senescence and transformation model, as well as for patient samples (450k array, patient data split into CGI hypermethylation prone and exceptions, see **Fig. 2**). Lines denote the median, edges denote the IQR, whiskers denote 1.5 × IQR and minima/maxima are represented by dots.

**b)** Boxplot showing the fraction of methylated CGIs that fall within the PRC2 target and pan-cancer hyper CGI sets for BJ senescence and transformation samples as well as for different tumor types (450k array, patient data split into CGI hypermethylation prone and exceptions, see **Fig. 2**). Lines denote the median, edges denote the IQR, whiskers denote 1.5 × IQR and minima/maxima are represented by dots.


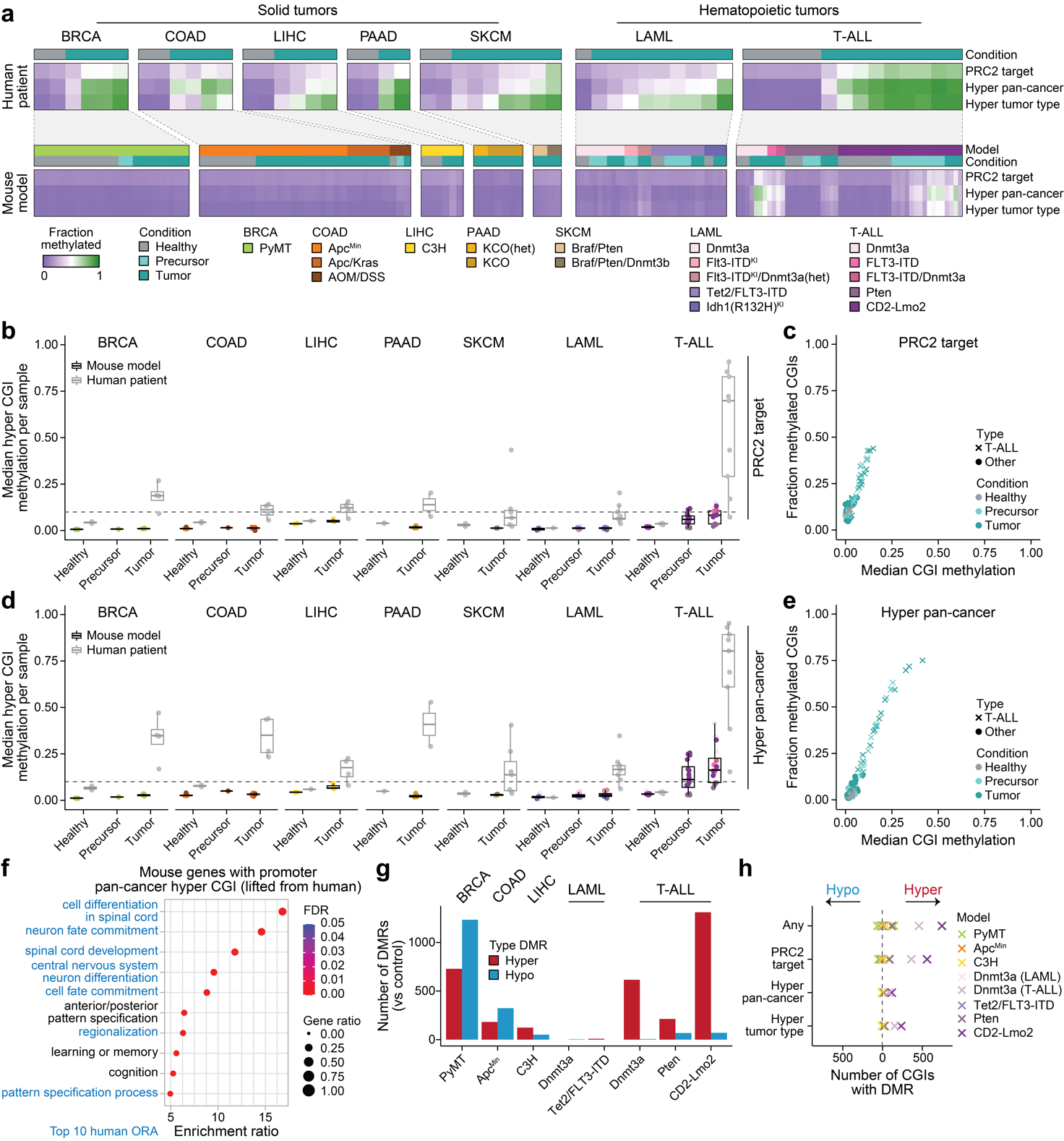


**Fig. S7: Differentially methylated regions between tumor and healthy tissue in mouse models**

**a)** Heatmaps showing the fraction of methylated PRC2 target CGIs, hypermethylated pan-cancer CGIs or tumor type-specific CGIs for human patients (top) and a large selection of genetically (and chemically) engineered mouse tumor models (bottom).

**b)** Boxplot showing the median methylation for PRC2 target CGIs for mouse models (black box) and human patients (grey box). Lines denote the median, edges denote the IQR, whiskers denote 1.5 × IQR and minima/maxima are represented by dots.

**c)** Scatterplot showing the median methylation and fraction of methylated CGIs of PRC2 target CGIs in healthy, precursor and tumor mouse model samples. T-ALL samples are marked with a cross.

**d)** Boxplot showing the median methylation across pan-cancer hyper CGIs for mouse models (black box) and human patients (grey box). Lines denote the median, edges denote the IQR, whiskers denote 1.5 × IQR and minima/maxima are represented by dots.

**e)** Scatterplot showing the median methylation and fraction of methylated CGIs of pan-cancer hyper CGIs for healthy, precursor and tumor mouse model samples. T-ALL samples are marked with a cross.

**f)** Overrepresentation analysis of mouse genes with promoters overlapping pan-cancer hyper CGIs (lifted from human, biological processes). Categories that are also enriched in the human set are marked in blue (**Fig. S1f**).

**g)** Number of DMRs between tumor and control samples for mouse models with at least three samples in each condition.

**h)** Number of CGIs overlapping hyper- or hypomethylated DMRs for the different mouse model comparisons. Overall, mainly T-ALL tumors are associated with larger numbers of CGIs overlapping hypermethylated DMRs.


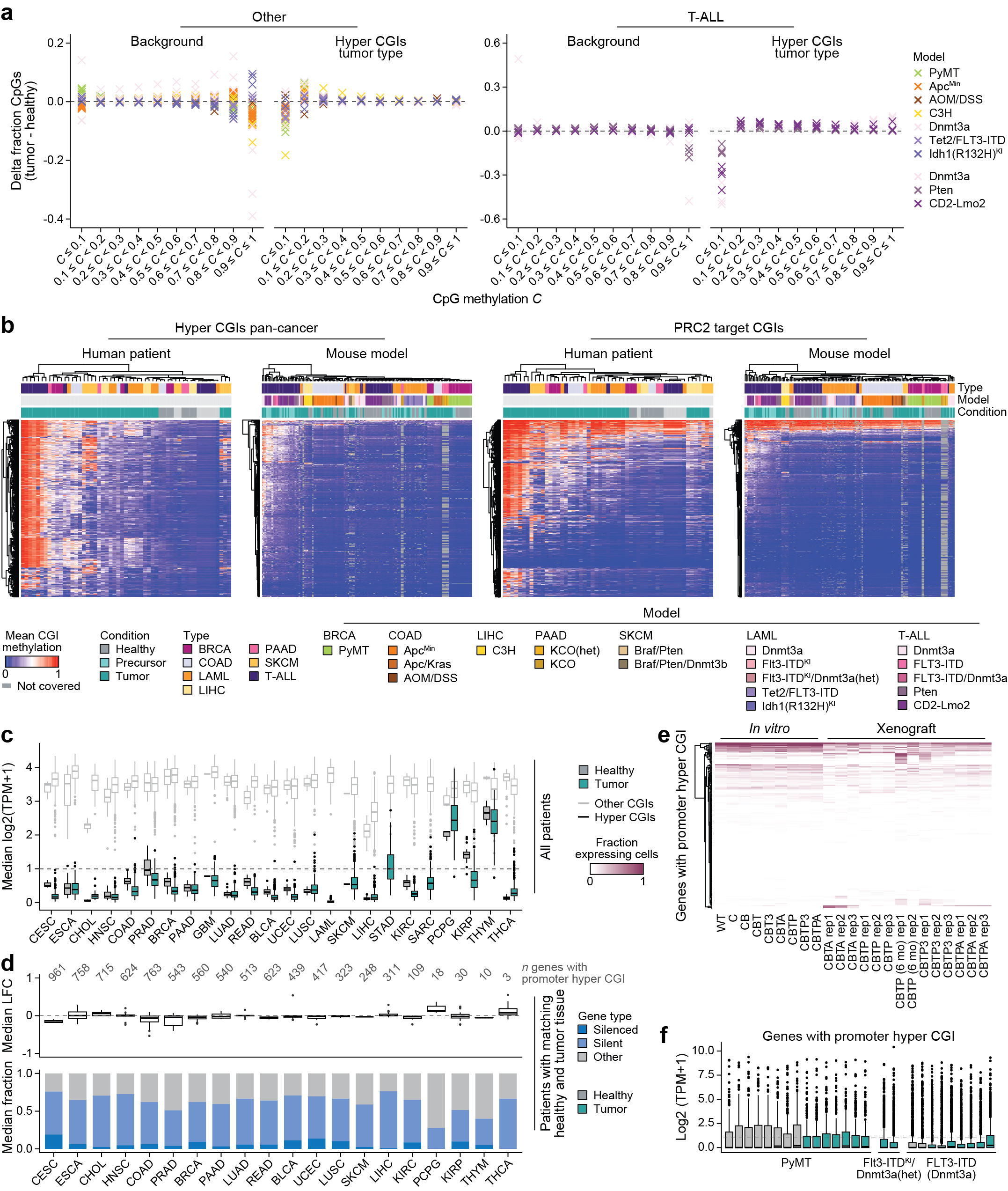


**Fig. S8: Transcriptional state of genes associated with hyper CGIs**

**a)**  Shifts in the global distribution of CpG methylation between tumor and matching healthy tissue, calculated as the difference in the fraction of CpGs that exist at different methylation levels. For the methylation distribution of individual samples, see **panels Fig. S8c-f**.

**b)** Heatmaps and hierarchical clustering of human patients or mouse models based on pan-cancer hyper CGIs (left) or PRC2 target CGIs (right). Although human patient samples cluster broadly based on condition (healthy/tumor), mouse models group mostly by tissue type/specific model (healthy and tumor samples combined), with the exception of the T-ALL samples that methylate subsets of cancer-associated CGIs and cluster as an outgroup.

**c)** Median log2-transformed expression across genes with promoter hyper CGIs (black outlined boxplots) and other CGIs with promoter CGIs (grey outlined boxplots) for each TCGA patient per tumor type (patients with available expression data are considered). Lines denote the median, edges denote the IQR, whiskers denote 1.5 × IQR and minima/maxima are represented by dots. The dotted line indicates a TPM of 1, which we consider as the cut-off between inactive and active genes.

**d)** Top: Median log2 fold change (LFC) of the expression of genes with promoter hyper CGI between tumor and healthy tissue from the same TCGA patient. Lines denote the median, edges denote the IQR, whiskers denote 1.5 × IQR and minima/maxima are represented by dots. Bottom: Categorization of genes into silent (TPM < 1 for both healthy and tumor tissue), silenced (TPM < 1 for tumor tissue and LFC < -1) and remaining genes.

**e)** Fraction of single cells that express (count > 0) genes with promoter hyper CGIs in the human melanoma model at different stages (*in vitro* and xenograft). Most genes are not expressed across wild type and mutated samples.

**f)** Log2-transformed expression of genes with promoter hyper CGIs in different mouse models (models with available RNA-seq data from the same studies or labs were chosen). The majority of genes associated with CGI hypermethylation in humans are not expressed from orthologous loci in the mouse. Lines denote the median, edges denote the IQR, whiskers denote 1.5 × IQR and minima/maxima are represented by dots. The dotted line indicates a TPM of 1, which we consider as the cut-off between inactive and active genes.
